# Overall Oxygen Electrocatalysis on Nitrogen‐Modified Carbon Catalysts: Identification of Active Sites and In Situ Observation of Reactive Intermediates

**DOI:** 10.1002/anie.202012615

**Published:** 2020-12-08

**Authors:** Yangming Lin, Zigeng Liu, Linhui Yu, Gui‐Rong Zhang, Hao Tan, Kuang‐Hsu Wu, Feihong Song, Anna K. Mechler, P. Philipp M. Schleker, Qing Lu, Bingsen Zhang, Saskia Heumann

**Affiliations:** ^1^ Max Planck Institute for Chemical Energy Conversion Stiftstrasse 34–36 45470 Mülheim an der Ruhr Germany; ^2^ Institut für Energie und Klimaforschung (IEK-9) Forschungszentrum Jülich GmbH 52425 Jülich Germany; ^3^ Ernst-Berl-Institut für Technische und Makromolekulare Chemie Technische Universität Darmstadt Alarich-Weiss-Strasse 8 64287 Darmstadt Germany; ^4^ National Synchrotron Radiation Laboratory University of Science and Technology of China Hefei 230029 P. R. China; ^5^ School of Chemical Engineering University of New South Wales Kensington, Sydney NSW 2052 Australia; ^6^ Beijing National Laboratory for Molecular Sciences Institute of Chemistry Chinese Academy of Sciences Beijing 100190 China; ^7^ Shenyang National Laboratory for Materials Science Institute of Metal Research Chinese Academy of Sciences Shenyang 110016 P. R. China

**Keywords:** active site, in situ infrared spectroscopy, intermediates, metal-free carbon, nitrogen doping

## Abstract

The recent mechanistic understanding of active sites, adsorbed intermediate products, and rate‐determining steps (RDS) of nitrogen (N)‐modified carbon catalysts in electrocatalytic oxygen reduction (ORR) and oxygen evolution reaction (OER) are still rife with controversy because of the inevitable coexistence of diverse N configurations and the technical limitations for the observation of formed intermediates. Herein, seven kinds of aromatic molecules with designated single N species are used as model structures to investigate the explicit role of each common N group in both ORR and OER. Specifically, dynamic evolution of active sites and key adsorbed intermediate products including O_2_ (ads), superoxide anion O_2_^−^*, and OOH* are monitored with in situ spectroscopy. We propose that the formation of *OOH species from O_2_^−^* (O_2_^−^*+H_2_O→OOH*+OH^−^) is a possible RDS during the ORR process, whereas the generation of O_2_ from OOH* species is the most likely RDS during the OER process.

## Graphical abstract

Dynamic evolution of active sites and key oxygen intermediate products during the ORR and OER on N‐doped carbon catalysts are monitored experimentally with in situ ATR‐IR spectra. With the assistance of isotopic labeling, the formation of *OOH species from O_2_^−^* (O_2_^−^*+H_2_O→OOH*+OH^−^) is suggested to be a possible RDS during the ORR process, whereas the generation of O_2_ from OOH* species is the most possible RDS during the OER process.

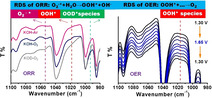

## Introduction

Overall oxygen electrocatalysis, including oxygen reduction reaction (ORR) and oxygen evolution reaction (OER), represents the cornerstone for a wide range of renewable electrochemical energy conversion and energy storage technologies.[^1^, ^2^, ^3^] Nitrogen (N)‐modified graphitic carbon materials, as the most promising metal‐free catalysts in these two reactions, have received tremendous attention over the last decade, owing to their abundance and high sustainability compared with metal catalysts.[^4^, ^5^] Some impressive experiments and theoretical predictions regarding the possible catalytic mechanisms and active sites have been conducted by regulating the N concentration in N‐modified model materials or introducing the simulation models of the active structures.[^6^, ^7^, ^8^] Although pyridinic N and graphitic N have recently been pointed out to be the most likely species that could create active sites, the connection remains controversial due to the inevitable mixing with other N configurations (e.g. pyrrolic, amine, or lactam) in doped carbon catalysts synthesized by the recent, deficient doping methods. Specifically, the involved intermediate products (e.g. adsorbed O_2_ molecule, superoxide anion O_2_^−^*, peroxide HO_2_*) and rate‐determining steps (RDS) during the ORR and OER processes have been rarely studied by experimental approaches. The precise structure–function relationship between active sites and reactivities is still unclear. The inhomogeneities of N species associated with the morphology of catalysts also hamper the exploration of active sites. Determining the genuine active sites, monitoring the important intermediate products, and revealing the possible reaction pathways is therefore highly important in order to understand the catalytic nature and to optimize the design and development of new carbon‐based catalysts.

**Figure 1:**
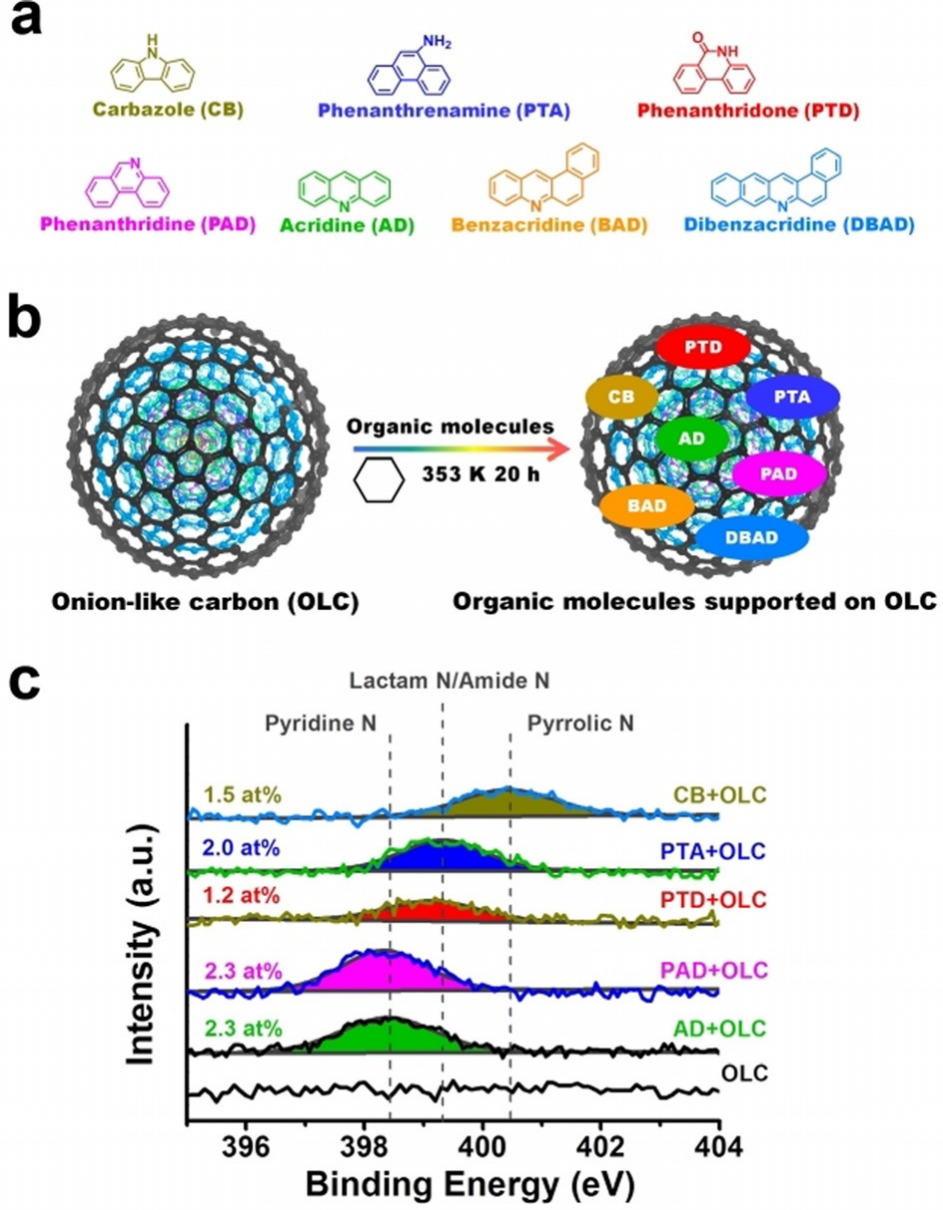
Structural, fabrication and elemental characterization of model catalytic systems. a) Structures of different aromatic organic molecules with isolated N configurations (including pyrrolic, amine, lactam, and pyridinic N). b) The preparation of aromatic molecules supported on OLC using a solvothermal method. Here, cyclohexane was used as the solvent. c) XPS N1s spectra of OLC and model catalytic systems. The FWHM of all N peaks is approximately 1.6 eV.

To disclose the genuine role of each N species in oxygen electrocatalysis, preparing N‐containing graphitic carbon materials with one single N species is ideal to provide direct inference; but the inductive influence of surface oxygen species and defect sites (heteroatom‐free) of carbon materials on various resultant N species is difficult to rule out. Alternatively, aromatic organic molecules have been considered as graphene molecules at the nanoscale due to their similar π‐conjugated structures and properties.^[9]^ A promising strategy is to use functionalized aromatic organic molecules (Figure 1 a) with isolated N species as model active matrices in order to mimic the edge structure of N‐doped carbon materials and to identify the active sites.[^10^, ^11^] The electronic and reactive properties of active components can be tuned by extending the π‐conjugated domains of these organic molecules at a discrete molecular level.^[10]^ Moreover, the intrinsic low conductivities and dispersibilities of these organic molecules can be improved by using nanocarbon materials as supports to build electrically coupled catalytic systems through non‐destructive π–π interactions.[^12^, ^13^]

Herein, three nanocarbon supports—onion‐like carbon (OLC), high temperature‐treated carbon nanofiber (HHT), and carbon nanotubes (CNTs)—were chosen to support seven different model aromatic organic molecules to form model catalytic systems via a solvothermal self‐assembly process (Figure 1 b, here, OLC is used as an example in the preparation process). The resulting model catalytic systems not only have a high concentration of desired single N species, but also exhibit a clear structure–activity relationship. The pyridinic N species are demonstrated to be advantageous for both ORR and OER processes over a wide pH range. The ORR is dominated by a process similar to the four‐electron‐like pathway, which is associated with the local conjugated structure of N species. The activities of catalysts mainly originate from electrocatalytic reactions rather than from carbon corrosion during the OER process. The possibly adsorbed intermediates and RDS are proposed for both ORR and OER processes.

## Results and Discussion

### Determination of a Model Catalytic System with a Single N Group

As shown in Figure 1 c, the results of X‐ray photoelectron spectroscopy (XPS) confirm main four N species: pyridinic N groups (398.3 eV), lactam or amide N groups (399.2 eV), or pyrrolic N groups (400.3 eV) exist in these model catalytic systems.^[14]^ Due to the good symmetry and the narrow full width at half‐maximum (FWHM, ca. 1.6 eV) of all N peaks, we can conclude that each model catalytic system contains only one type of N functional group. The content of N species on the model systems ranges from 1.2 to 2.3 at %. The structural features of these model molecules on OLC support were further studied by attenuated total reflectance infrared (ATR‐IR) spectroscopy (Figure S1–S5). The main characteristic peaks of CB, PTD, PTA, PAD, and AD molecules can be clearly observed, meaning that the structures of various model molecules are maintained during the preparation process. The net contents of these model molecules on OLC support are quantified to be 0.72–1.31 wt % by using thermogravimetric (TG) measurements (Figure S6).

### Model Catalysts Reveal ORR Active Sites

**Figure 2:**
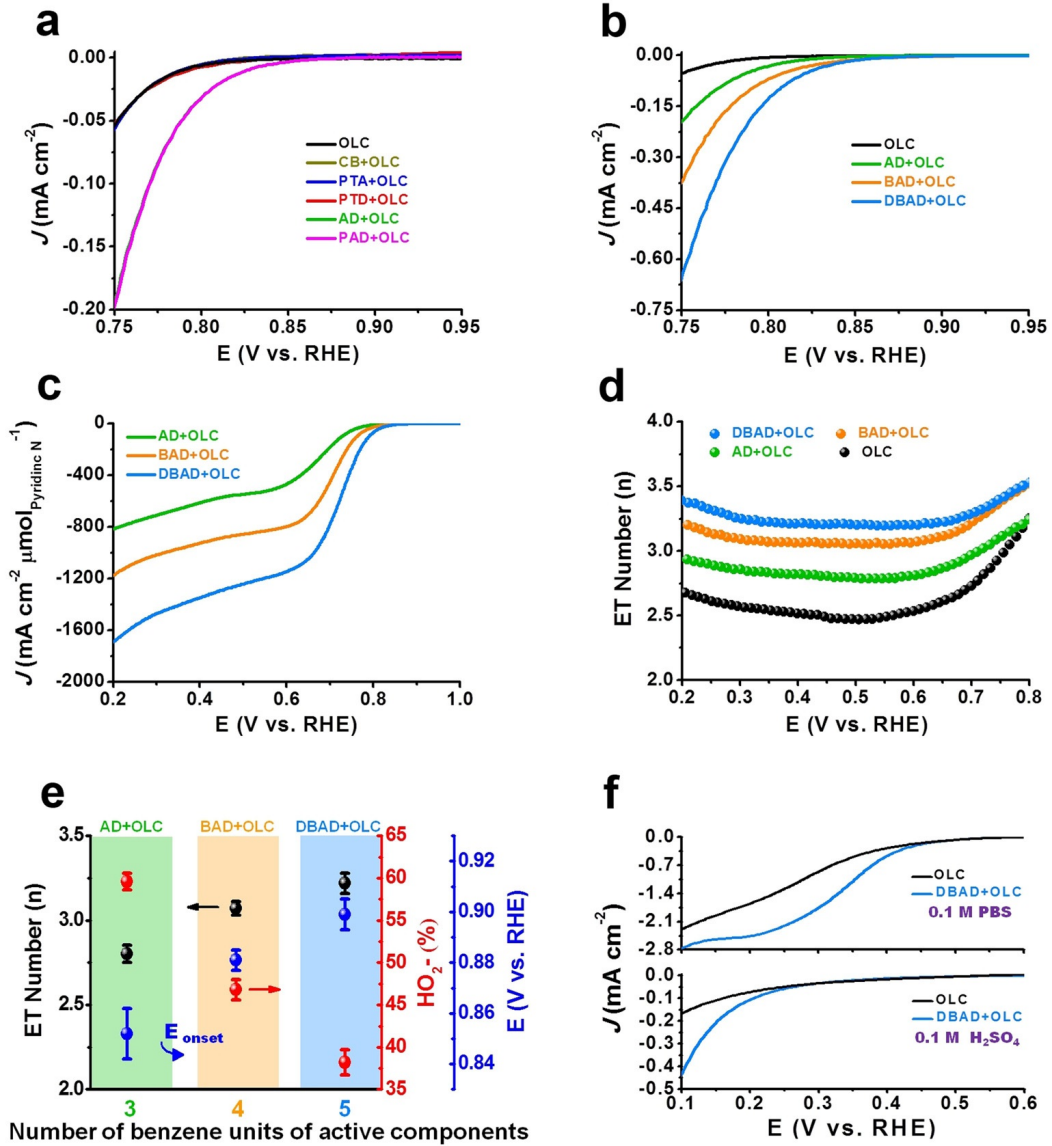
Electrochemical measurements of model catalytic systems for the ORR in O_2_‐saturated electrolytes. a) LSV curves of model catalytic systems and pure OLC catalyst in 0.1 m KOH and b) model catalytic systems bearing isolated pyridinic N species and extended π‐conjugated structure at onset potential region. c) The intrinsic current densities of various model catalytic systems with isolated pyridinic N species. d) Electron transfer (ET) numbers of supported catalysts with isolated pyridinic N species and pristine OLC catalyst, respectively, based on rotating ring‐disk electrode (RRDE) measurements. e) Number of benzene units of active components with single pyridinic N species as a function of ET number, HO_2_^−^ selectivity at 0.6 V_RHE_, and onset potential (*E*_onset_). Here, *E*_onset_ is defined by a threshold current density of 1 μA cm^−2^. f) LSV curves measured in phosphate buffer solution (pH=7.0) and 0.1 m H_2_SO_4_ media, respectively.

The ORR activity of these model catalytic systems in alkaline medium is shown in Figure 2 a. Only supported AD (AD+OLC) and PAD (PAD+OLC) model catalytic systems exhibit higher current densities and more positive onset potentials (*E*_onset_) compared with pure OLC and other supported model systems with respective pyrrolic N (CB+OLC), O=C−NH (PTD+OLC) and −NH_2_ (PTA+OLC) groups, while unsupported model molecules do not show the relevant activities (Figure S7), indicating that pyridinic N species are the possible active species in the ORR. To further investigate the role of pyridinic N species, two other concentrations of AD molecules in AD+OLC are deliberately regulated (Figure S6f). It can be found that the current density of AD+OLC is declining with decreased concentrations of AD molecules (Figure S8a,b), reflecting the important role of pyridinic N species. Furthermore, the similar behavior of AD+OLC and PAD+OLC catalysts indicates that the position of the pyridinic N, noted as the edge zigzag or armchair position, negligibly affects catalytic performance. Anthracene (AT) was additionally used to mimic the zigzag configuration of pure carbon materials (Figure S9). The lower performance of supported AT (AT+OLC) relative to AD+OLC further indicates the activity enhancement of pyridinic N species as well as the non‐critical role of edge configuration in the ORR process in alkaline medium.

To investigate the influence of the size of the π‐conjugated structure for the pyridinic N species on the ORR activity, two other molecules, BAD and DBAD, were chosen. The net concentrations of these two molecules are determined to be 1.18 and 1.05 wt %, respectively (Figure S10). Their molecular structures exhibit good stability as confirmed by ATR‐IR (Figures S11 and S12). In the C K‐edge X‐ray absorption near‐edge structure (XANES) spectra (Figure S13a), compared with pristine OLC, an additional peak of supported DBAD (DBAD+OLC), observed at 287.2 eV, can be assigned to the σ* bond of C−N.^[15]^ Another weak peak appeared at about 286.6 eV for DBAD+OLC which might be attributed to π* (ring) resonance of the DBAD molecule itself.^[16]^ Other carbon–nitrogen species could not be observed. In the N K‐edge XANES spectra (Figure S13b), compared with pristine OLC, DBAD+OLC exhibits an obvious peak at 398.3 eV which can be assigned to a π* feature of a pyridine‐like N structure,^[17]^ suggesting that the dominant N species on the surface of OLC is the expected pyridinic N group. This is further supported by XPS results of DBAD+OLC (Figure S16b), which shows that only pyridinic N species (at 398.4 eV, 1.8 at %) can be observed. The LSV results displayed in Figures 2 b and S14a show that the current densities are directly proportional to the sizes of the π‐conjugated structures with pyridinic N species. Further electrocatalytic results summarized in Figure 2 c,e demonstrate that the size of the π‐conjugated system is a critical parameter to accelerate the ORR process; this is indicated by the gradually enhanced intrinsic current densities and the gradual positive shift in onset potential (*E*_onset_), which might be ascribed to the enhanced electron delocalization effect from a larger π‐conjugated system of model molecules. In addition, the reasonable catalytic stability of DBAD+OLC at two applied potentials was demonstrated by the absence of a significant current change for 2 h (Figure S16a). To further confirm the role of pyridinic N species, DBAD was removed from DBAD+OLC (Figure S15) by ultrasonication. The corresponding activity of the residual OLC sample returns to the level of pristine OLC, which evidences the advantageous role of pyridinic N species for ORR activity.

The introduction of pyridinc N species significantly increases the electron transfer (ET) number from 2.4–2.7 for OLC to 2.8–3.0 for AD+OLC and can be further extended to 3.3–3.5 for DBAD+OLC at 0.2–0.7 V vs. RHE (V_RHE_) (Figure 2 d,e). The corresponding selectivity in favor of HO_2_^−^ production gradually decreases from 70–78 % for OLC to 50–60 % for AD+OLC, and eventually reaches 19.0–38.0 % for DBAD+OLC at 0.2–0.7 V_RHE_ (Figure S14b). Moreover, as a comparison, an AT+OLC sample with single‐edge configuration (zigzag) and without any pyridinic N species displays higher HO_2_^−^ selectivity than OLC and AD+OLC (Figure S9b). All these findings indicate that (i) pyridinic N species are prone to contribute the ORR in a four‐electron‐like manner (as illustrated by the increase in ET number and decrease in HO_2_^−^ production); (ii) pyridinic N species could improve the *E*_onset_ of ORR to more positive values; (iii) the structure size of π‐conjugated pyridinic N species is a critical factor to facilitate the ORR process; and (iv) the edge structure in the absence of pyridinic N species (for example, only zigzag configuration) has a negative effect on the acceleration of the four‐electron pathway of ORR. Therefore, it can be expected that carbon‐based catalysts with a single pyridinic N function group in a larger π‐conjugated system (e.g. nanographene) could show a complete four‐electron pathway.

Additionally, DBAD+OLC is selected as a representative catalyst to study the role of pyridinic N in neutral (0.1 m phosphate buffer solution, pH=7.0) and acidic (0.1 m H_2_SO_4_) ORR processes. This catalyst delivered a higher current density than pristine OLC in neutral and acidic media (Figure 2 f), which suggests that the model catalyst containing pyridinic N species can exhibit an elevated catalytic performance over a broad pH range.

### Model Catalysts Reveal OER Active Sites

**Figure 3:**
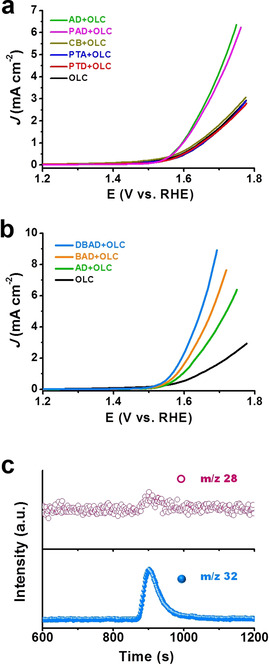
Electrochemical measurements of model catalytic systems for the OER in Ar‐saturated 0.1 m KOH. a) LSV curves of model catalytic systems and pristine OLC catalyst. b) LSV curves of model catalytic systems with isolated pyridinic N species and extended π‐conjugated structure catalysts. c) Mass spectra profiles of the produced CO (*m*/*z* 28) and O_2_ (*m*/*z* 32) with DBAD+OLC.

In addition to the identification of active sites for ORR, we also unveiled the catalytic function of each N species for the OER activity by evaluating the model catalytic systems in 0.1 m KOH (Figures 3 and S17). Similar to the ORR results, AD+OLC and PAD+OLC catalysts show higher current densities than other catalysts. This indicates that pyridinic N species are also active for OER. In contrast, pyrrolic, amine, and lactam species are inactive for the OER process, as demonstrated by the insignificantly improved performance of CB+OLC, PTA+OLC and PTD+OLC relative to OLC (Figure 3 a). Also, in line with the ORR results, the OER process is insensitive to the edge configurations as demonstrated by the similar current densities and Tafel slopes of AD+OLC and PAD+OLC catalysts (Figures 3 a and S18). By extending the π‐conjugated system of the model molecules, the OER activity of the catalysts can be increased (Figure 3 b). There is a directly proportional relationship between the number of benzene units and theoretical TOF values, as shown in Figure S19. Specifically, the value increases from 0.153 s^−1^ for AD+OLC to 0.638 s^−1^ for DBAD+OLC at 1.60 V_RHE_. Both values are significantly higher than that of the reported highly active Ni/Fe‐based OER catalysts (0.028–0.075 s^−1^ at 1.63 V_RHE_).[^18^, ^19^] Hence, it can be concluded that the π‐conjugated size of the carbon matrix accelerates the OER process. Moreover, similar Tafel slopes (77–85 mV dec^−1^) are obtained for AD+OLC, BAD+OLC, and DBAD+OLC catalysts (Figure S20), which implies that the extended π‐conjugation does not change the OER reaction pathway. By using the rotating ring‐disk electrode (RRDE) technique (Figure S21), the OER process occurring on DBAD+OLC is dominated by a desirable four‐electron pathway (99.9 %) with negligible peroxide intermediate formation.

A high Faradaic efficiency (FE(O_2_)) of around 86 % at two low potentials (e.g. 1.567 and 1.60 V_RHE_) was obtained, which are comparable to reported metal‐based catalysts to some degree.[^20^, ^21^] The loss of FE(O_2_) may be partly due to the inefficient oxygen collection by the Pt ring electrode or unavoidable carbon corrosion (e.g. CO or CO_2_ formation) in alkaline medium. It should be emphasized that, when acquiring mass spectra, a very weak CO signal and an obvious O_2_ signal were observed, as shown in Figure 3 c, reflecting that the current density originates mainly from water oxidation rather than carbon corrosion. DBAD+OLC shows a decent stability during 2 h at an applied potential (Figure S16a). All the aforementioned results suggest that pyridinic N species can efficiently accelerate both ORR and OER processes, and their catalytic performance could be regulated by the π‐conjugated size of active components at the molecular level. To further verify the catalytic role of pyridinic N species, two other kinds of carbon (CNT and HHT) were tested as supports for DBAD. Indeed, dramatically enhanced catalytic behavior can be observed with both DBAD+HHT and DBAD+CNT catalysts for the ORR and OER processes (Figures S22 and S23), which confirms the critical role of pyridinic N species in facilitating the catalytic processes.

### Doped Catalysts for OER and ORR

**Figure 4:**
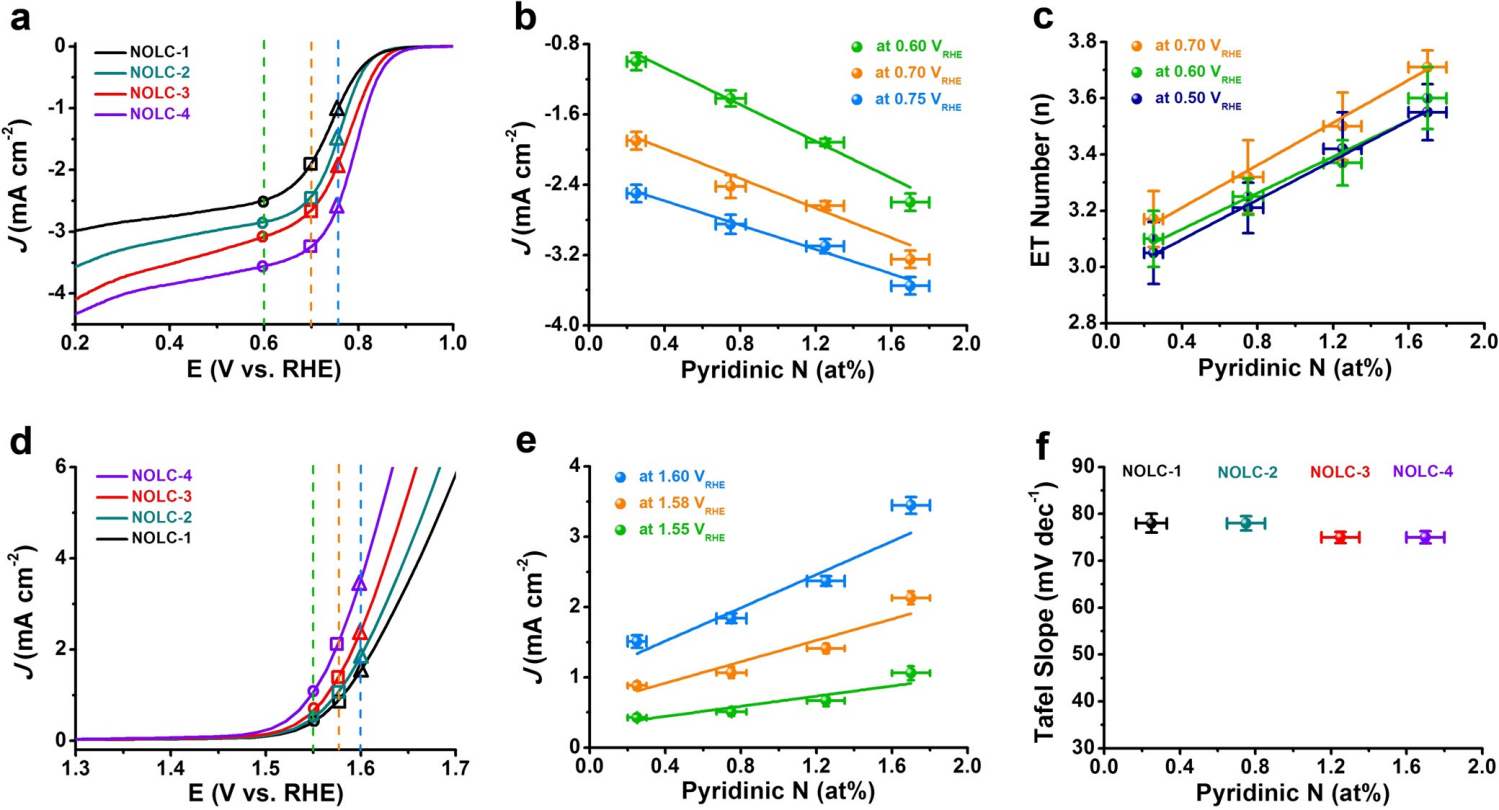
Electrochemical measurements of various N‐doped OLC (NOLC) catalysts with enriched pyridinic N and without any graphitic N species for ORR and OER. a) ORR LSV curves of NOLC catalysts. b,c) Dependence of current densities and ET numbers at different potentials on the concentration of pyridinic N of NOLC catalysts (based on XPS results). d) OER LSV curves of NOLC catalysts. e,f) Dependence of current densities at different potentials and Tafel slope on the concentration of pyridinic N (based on XPS results).

In general, pyridinic N and graphitic N are considered to be the most common species in oxygen electrocatalysis with N‐doped carbon as catalysts.[^8^, ^22^, ^23^] After confirming the positive role of pyridinic N species with model molecules, four kinds of N‐doped OLC (NOLC) catalysts with enriched pyridinic N but without any graphitic N species were synthesized and tested for both ORR and OER processes (Figure S24). Among them, NOLC‐4 possesses the highest N content (2.7 at %) and largest proportion (63.1 %) of pyridinic N species in all N species. As shown in Figure 4, by varying the contents of pyridinic N species in different NOLC catalysts, the current densities and onset potentials can be gradually manipulated for the ORR activity, while only the current densities are impacted for the OER activity. Some linear relationships between current densities or ET numbers and the concentrations of pyridinic N species at different applied potentials (Figure 4 b,c,e) could be observed in the ORR and OER processes, reflecting the fact that pyridinic N species are the catalytically active sites in ORR and OER processes. Moreover, the Tafel slope values (Figures 4 f and S25, 75–78 mV dec^−1^) of NOLC catalysts are analogous to the model catalytic systems (77–85 mV dec^−1^), suggesting that both model catalysts and doped catalysts have similar kinetic reaction processes.

### Mechanistic ORR and OER Studies

**Figure 5:**
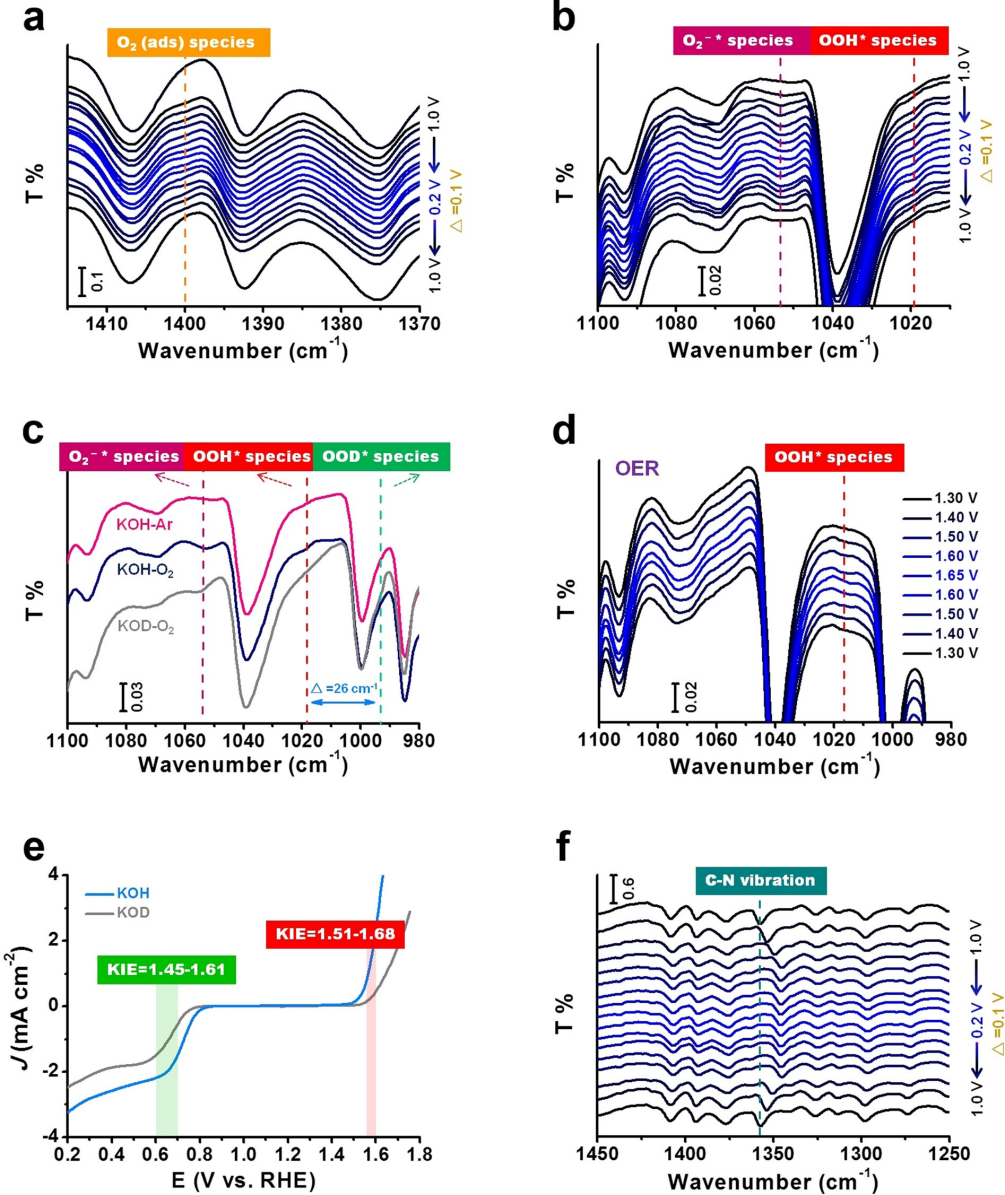
In situ ATR‐IR spectra for monitoring dynamic evolution of the involved intermediate oxygen species, determining the rate‐determining steps (RDS) and identifying the active sites on DBAD. a–c,f) ORR and d) OER processes. The experiments including Ar‐saturated KOH, O_2_‐saturated KOH, and O_2_‐saturated KOD were carried out at 0.6 V_RHE_. Here, O_2_^−^*, OOH*, and OOD* represent the adsorbed O_2_^−^, HO_2_, and DO_2_ species, respectively. e) Isotopic electrochemical studies of representative DBAD+OLC catalyst in 0.1 m KOH and 0.1 m KOD.

Density functional theory calculations predict that the binding energy of some surface adsorbed intermediate oxygen species, such as O_2_ (ads), O_2_^−^*, OOH*, O*, and OH*, governs the ORR activity,[^24^, ^25^, ^26^] whereas OH*, O*, and OOH* mainly dominate the OER process.^[27]^ It is important to use effective experimental measurements to elucidate the evolution processes of surface‐relevant species during electrochemical reactions. As shown in the in situ ATR‐IR spectra in Figure 5 a, the peak at 1400 cm^−1^ exhibits a quasi‐reversible behavior with ORR potential change, which is attributed to the O−O vibration of the adsorbed oxygen molecule O_2_(ads) on the catalyst.^[28]^ Moreover, evolution processes monitored by two other peaks at 1052 and 1019 cm^−1^ are observed (Figure 5 b). Given that the infrared vibration peaks of O_2_^−^* and OOH* typically are located in the region 1100–980 cm^−1^,[^28^, ^29^, ^30^, ^31^, ^32^, ^33^] some control experiments were carefully introduced to further differentiate O_2_^−^* and OOH* species. The results of Figure 5 c reveal that (i) no obvious peaks at 1052 and 1019 cm^−1^ positions can be observed in Ar‐saturated KOH, suggesting these two peaks correspond to O‐containing species, and (ii) an apparent red‐shift behavior (Δ≈26 cm^−1^) appears at 1019 cm^−1^ for O_2_‐saturated KOD electrolyte, while the original vibration band at 1052 cm^−1^ is unaffected (Figures 5 c and S26), indicating that the vibration band at 1019 cm^−1^ is most likely OOH* rather than O_2_^−^*, thus the vibration band at 1052 cm^−1^ is assigned to O_2_^−^*. All experiments demonstrate O_2_^−^* and OOH* species to be the important intermediates involved in the ORR process. Recently, the formation of O_2_^−^* from O_2_ (ads) coupled with the first electron transfer (that is, O_2_+e^−^→O_2_^−^*) or the generation of OOH* from O_2_^−^* (O_2_^−^*+H_2_O→OOH*+OH^−^) over carbon catalysts in the ORR has been considered to be the most common RDS.[^34^, ^35^] To elucidate the RDS, isotopic electrochemical studies were performed, as displayed in Figure 5 e. Kinetic isotope effect (KIE) values of around 1.45–1.61 at 0.6–0.7 V_RHE_ are obtained, suggesting that there is a potential primary isotope effect and the RDS of the ORR process involves a cleavage of the O−H bond of water molecules. Combined with the ATR‐IR results, the following elementary step O_2_^−^*+H_2_O→OOH*+OH^−^ is likely to be the potential RDS in the ORR. In the case of the OER, a similar evolution behavior of OOH* species (at 1018 cm^−1^) is observed and no other oxygen species can be found in Figure 5 d, implying that the RDS in the OER is mainly associated with the OOH* species. Together with the result of the primary isotope effect with the KIE values of around 1.51–1.68 at 1.567–1.6 V_RHE_ (Figure 5 e), this allows us to conclude that the generation of O_2_ from OOH* species is the most possible RDS in the OER.

To gain more insight into the active sites, C−N vibrational bands were monitored with in situ ATR‐IR spectroscopy. The red‐shift of the C−N peak at 1357 cm^−1^ (Figures 5 f and S27) with a potential‐dependent tendency suggests that active sites should originate from pyridinic N itself or adjacent carbon atoms during the ORR and OER. To determine the real active sites, some post‐mortem measurements were further conducted after ORR and OER stability tests. The XPS spectrum of DBAD+OLC after ORR (Figure S16b) shows that the concentration of pyridinic N decreases and a significant new N1s peak at 400.1 eV appears, which can be attributed to pyridonic N resulting from pyridinic N through a reaction involving the carbon atoms next to pyridinic N‐ and O‐containing species (possibly OH or OOH).^[6]^ Therefore, it seems that the active sites are the adjacent carbon atoms of the pyridinic N species rather than pyridinic N itself. Similar to the case of ORR, an impressive pyridonic N peak appears in the XPS N1s spectrum after OER operation (Figure S16b), which also implies that the active sites are likely the adjacent carbon atoms of the pyridinic N species. Additionally, we do not observe any characteristics of pyridine N‐oxide species at approximately 402 eV in the N1s peak of XPS and at circa 1265 cm^−1^ in the ATR‐IR spectra during the ORR and OER.^[36]^ Thus, we propose that the neighboring carbon atoms are strongly involved in the acceleration of both ORR and OER processes.

## Conclusion

We have gained experimental insight into the catalytic mechanisms at a molecular level using aromatic organic molecules with designated N species as models. Pyridinic N species play a crucial role for the electrochemical ORR over a wide pH range and alkaline OER. It can be concluded that pyridinic N species are prone to facilitate the ORR process by a four‐electron‐like pathway, and they also improve the catalytic activity rather than carbon corrosion for the OER. Furthermore, the location at edge zigzag or armchair positions of pyridinic N does not obviously affect the catalytic performance. The structure–function relationship of the active component can be established by delicately controlling their longitudinal extension (π‐conjugated structures) and edge configurations. We believe that OOH* (that is, HO_2_*) and/or O_2_^−^* species are involved as the key intermediates in both ORR and OER processes, which was corroborated by in situ ATR‐IR spectra and isotopic labeling studies. Neighboring carbon atoms of pyridinic N species are likely the active sites, as demonstrated by the dynamic evolution of the vibration peaks. To the best of our knowledge, this work for the first time provides spectral evidence of the dynamic evolution of key intermediate products and the RDS of metal‐free carbon catalysts in the overall oxygen electrocatalysis at a molecular level.

## Conflict of interest

The authors declare no conflict of interest.

## Supporting information

As a service to our authors and readers, this journal provides supporting information supplied by the authors. Such materials are peer reviewed and may be re‐organized for online delivery, but are not copy‐edited or typeset. Technical support issues arising from supporting information (other than missing files) should be addressed to the authors.

Supplementary
