# Supplementary material for: Overall Oxygen Electrocatalysis on Nitrogen‐Modified Carbon Catalysts: Identification of Active Sites and In Situ Observation of Reactive Intermediates
Source: Angew Chem Int Ed Engl. 2020 Dec 8;60(6):3299–306. doi: 10.1002/anie.202012615 (PMC7898341; doi:10.1002/anie.202012615)
Supplement: Supplementary file 1 — Supplementary [file ANIE-60-3299-s001.pdf]

Angewandte Chemie
Eine Zeitschrift der Gesellschaft Deutscher Chemiker

Supporting Information

# Overall Oxygen Electrocatalysis on Nitrogen-Modified Carbon Catalysts: Identification of Active Sites and In Situ Observation of Reactive Intermediates

*Yangming Lin,* Zigeng Liu, Linhui Yu, Gui-Rong Zhang, Hao Tan, Kuang-Hsu Wu, Feihong Song, Anna K. Mechler, P. Philipp M. Schleker, Qing Lu, Bingsen Zhang, and Saskia Heumann**

anie_202012615_sm_miscellaneous_information.pdf

# Supplementary Materials

.

**This PDF file includes:**

Experimental section

Figure S1 to S29

# Table of Contents

**Figure S1.** Attenuated total reflectance infrared (ATR-IR) spectra of pure CB, OLC and CB+OLC samples.

**Figure S2.** ATR-IR spectra of pure PTD, OLC and PTD+OLC samples.

**Figure S3.** ATR-IR spectra of pure PTA, OLC and PTA+OLC samples.

**Figure S4.** ATR-IR spectra of pure PAD, OLC and PAD+OLC samples.

**Figure S5.** ATR-IR spectra of pure AD, OLC and AD+OLC samples.

**Figure S6.** TG analysis of pure OLC and different model catalytic systems.

**Figure S7.** Electrochemical measurements of various pure N-containing organic molecules and pristine OLC catalysts for the ORR in 0.1 M KOH.

**Figure S8**. **Electrochemical measurements of AD+OLC with different concentration of AD molecules for the ORR in 0.1 M KOH.**

**Figure S9.** Electrochemical measurements of OLC, AT+OLC and AD+OLC for the ORR in 0.1 M KOH.

**Figure S10.** TG analysis of pristine OLC, BAD + OLC and DBAD + OLC samples.

**Figure S11.** ATR-IR spectra of pure BAD, OLC and BAD+OLC samples.

**Figure S12.** ATR-IR spectra of pure DBAD, OLC and DBAD+OLC samples.

**Figure S13.** X-ray absorption near-edge structure (XANES) spectra (incident angle θ=45o) of representative pristine OLC, DBAD+OLC samples.

**Figure S14.** Electrochemical measurements of model catalytic systems consisting of pyridinic N-containing organic molecules and OLC support for the ORR in 0.1 M KOH.

**Figure S15.** Catalytic activity of pure OLC, DBAD+OLC and removed DBAD+OLC catalysts.

**Figure S16.** Stability tests and XPS spectra of DBAD+OLC catalyst.

**Figure S17.** LSV curves of pure N-containing organic molecules and pure OLC for the OER in 0.1 M KOH.

**Figure S18.** Tafel slopes of AD+OLC and PAD+OLC catalysts at a low working potential region.

**Figure S19**. Number of benzene units of active components with single pyridinic N species as a function of theoretical TOF at 1.6 $V_{RHE}$.

**Figure S20.** Tafel slopes of model catalytic systems consisting of pyridinic N-containing organic molecules and OLC support at a low working potential region.

**Figure S21.** Detection of $H_2O_2$ and $O_2$ evolution from DBAD+OLC catalyst applying RRDE measurements.

**Figure S22.** Electrochemical activities of DBAD+HHT and pure HHT catalysts.

**Figure S23.** Electrochemical activities of DBAD+CNT and pure CNT catalysts.

**Figure S24.** XPS spectra of various N-doped OLC (NOLC) catalysts without graphitic N (GN) species.

**Figure S25.** Tafel slopes of various NOLC catalysts at a low working potential region.

**Figure S26**. *In situ* ATR-IR spectra monitoring for OOH* and OOD* species at specific range (980 $cm^{-1}$-1040 $cm^{-1}$) during the ORR process.

**Figure S27.** *In situ* ATR-IR spectra monitoring for C-N vibration of DBAD active component during the OER process.

**Figure S28.** Tafel slopes of DBAD+OLC catalyst.

**Figure S29.** The in-situ ATR-IR schematic.

# Methods

### Materials

Acridine (AD) was purchased from Alfa. Carbazole (CB) and phenanthridinone (PTD) were supplied by TCI. KOD, 9-phenanthrenamine (PTA), phenanthridine (PAD), benz[a]acridine (BAD) and dibenz[a,i]acridine (DBAD) were obtained from Sigma-Aldrich. All reagents were used without further purification. Cyclohexane was purchased from Merck Co. (Germany). Purified ultra-dispersed nanodiamond (UDD) was bought from Beijing Grish Hitech Co. (China), produced by detonation and followed by acid washing. The surface area of OLC is about 460 $m^2/g$. Multi-walled carbon nanotubes (CNTs) were supplied by Shandong Dazhan Co. (China). The surface area of CNTs is about 230 $m^2/g$. HHT was bought from Pyrograf Products, Inc. The surface area of HHT is about 34 $m^2/g$ and it has the extreme low defect ratio ($I_D/I_G$=0.35). The latter two carbon materials were purified in concentrated HCl at room temperature for 20 h to remove the metal impurities before use.

### Synthesis of onion-like carbon (OLC)

OLC with fully graphite-like shell structures was prepared by annealing purified UDD at 1500 °C for 30 min in argon atmosphere. The yield of OLC was about 75%.

**Synthesis of model catalytic systems consisting of aromatic molecules and OLC, HHT or CNTs support**

In a typical procedure, 100 mg carbon support and 5 mg of model organic molecules, such as CB, PTD, PTA, PAD, AD, BAD and DBAD, were dissolved in 50 mL of cyclohexane to form a mixture which was then sonicated for 30 min under the room temperature. The obtained mixture was then transferred into a pressure bottle with a capacity of 150 mL and 900 rpm rotation rate and maintained at about 80 °C for 20 h. The final products were filtered and washed with a large amount of ethanol to remove the excess of model small molecules and cyclohexane and then dried them at 60 °C for 12 hours. The as-prepared supported catalysts were labeled as CB+OLC, PTD+OLC, PTA+OLC, PAD+OLC, AD+OLC, BAD+OLC and DBAD+OLC, respectively. Pure OLC was treated by the same process without the introduction of model catalysts and acted as reference materials. In the present work, the treated OLC without any molecules was also labeled as OLC. The supported DBAD on CNTs and on HHT samples were prepared by using the same procedure.

**Preparation of N-doped OLC (NOLC) catalysts with enriched pyridinic N and without any graphitic N (GN) species**

0.1 g of as-prepared OLC was directly calcined at 300°C, 350 °C, 400 °C and 450 °C for 8 h under an ammonia atmosphere, and the final products were noted as NOLC-1, NOLC-2, NOLC-3 and NOLC-4, respectively.

**Material Characterization**

Thermogravimetric (TG) analysis was performed on a NETZSCH STA449 F3 thermal analyzer. The atmosphere was argon, and the heating rate was 10 K/min starting from 40 °C to 900 °C. X-ray

photoelectron spectroscopy (XPS) was performed on an ESCALAB 250 XPS system with a monochromatized Al Kα X-ray source, and the first one scan was collected. Fourier transform infrared (FTIR) spectra were collected with a Thermo Scientific Nicolet iS50 in attenuated total reflectance infrared (ATR-IR) mode with a resolution of 4 $cm^{-1}$ at room temperature. Mass spectra measurement were performed on an OmniStarTM GSD 320 mass spectrometer purchased from PFEIFFER VACUUM Ltd. The detector type is C-SEM/Faraday. The carry gas was Ar with a flow rate of 1 mL/s. The $O_2$ was collected after 12 h at a constant potential of 1.6 V vs RHE ($V_{RHE}$) using a home-made cell with glass carbon pellet (Φ=1 cm) as a working electrode. The loading of DBAD+OLC catalyst on electrode are 0.5 $mg/cm^2$. In the case of *in situ* ATR-IR experiments, the investigated samples were dropcoated directly on the tip of the ATR unit, no binder was used. A KOH drop was deposited on top where a Pt wire (Φ=2 mm) and a leak-free Ag/AgCl (Φ=1 mm) electrode were used as counter electrode and reference electrode, respectively. All potentials were calibrated with respect to RHE scale according to the Nernst equation ($E_{RHE} = E_{Ag/AgCl} + 0.0592 \times pH + 0.197$ V). The operation time of each potential will be kept 2 min to ensure the proceeding of ORR or OER on catalyst. Then, the ATR data were collected with 16 scans at each applied potential. Each single spectrum will take around 15 s. The total running time of each potential is around 2 min and 15 s. The detail of in-situ ATR-IR schematic can be found in **Figure S29**. Chronoamperometry mode (Δ=0.05 or 0.1 V) was used to control the potential during *in situ* ATR-IR experiments. The XPS measurements for DBAD+OLC catalyst after ORR and OER processes used the pellet strategy. First, DBAD+OLC was pressed to pellets (Φ=6 mm) without using binder and then reacted in an $O_2$- or Ar-saturated 0.1 M KOH for 2 h at 0.60 and 1.60 $V_{RHE}$ for ORR and OER, respectively.

**Electrochemical measurements**

Electrochemical measurements were performed using a potentiostat/galvanostat (BioLogic VSP, France) with a three-electrode glass cell and rotator from PINE instruments. ORR experiment was carried

out in a rotating ring-disk electrode (RRDE) configuration consisting of a glassy carbon electrode (GCE) disk and a Pt ring electrode. The disk diameter is 5.61 mm, the Pt ring has an inner and outer diameter of 6.25 mm and 7.92 mm, respectively. The current collection efficiency is 37%. The modified samples were transferred onto the working electrode according to the following procedure: (1) 5 mg catalysts were added into the mixed solution of 960 μL $H_2O$, 4 mL isopropanol and 40 μL Nafion (binder), and then ultrasonicated for 10 min. (2) 12.6 μL of the ink was dropped on the GCE surface and dried at 40 °C for a few minutes in air. The final loading of catalyst on the electrode is about 0.051 mg/cm$^2$. The activities of the electrocatalysts were evaluated by the linear sweep voltammetry (LSV) sweeping from 1.0 V to 0.2 $V_{RHE}$ in an $O_2$- or Ar-saturated 0.1 M KOH and maintained at a rotation rate of 1600 rpm and 5 mV s$^{-1}$ scan rate. All LSV results were corrected by subtracting background experiments operated at Ar-saturated medium.

The $HO_2^-$ intermediate production percentages (% $HO_2^-$) were determined as follows:

$$\%HO_2- = \frac{200 I_r}{I_d N + I_r}$$

where $I_d$ is the disk current, Ir is the ring current and N is the current collection efficiency (37%).

The electron transfer (ET) number was calculated as follows:

$$n = \frac{4|I_{disk}|}{|I_{disk}| + I_{ring} / N}$$

In case of the OER experiment, a Pt wire was used as counter and a reversible hydrogen electrode (RHE, HydroFlex, Gaskatel GmbH) as reference electrode. For OER measurements, a simple rotating disk electrode (RDE) with a GC tip (5 mm diameter, 0.196 cm$^2$) was used as a working electrode. It was polished with 0.1 μm and 0.05 μm alumina powder and rinsed with deionized water, followed by sonication in ethanol and deionized water. Similarly, the final loading of the catalyst on the electrode is

about 0.051 mg/cm$^2$. OER polarization curves were obtained by LSV scanning from 1.0 $V_{RHE}$ to 1.8 $V_{RHE}$ with a scan rate of 5 mV/s in Ar-saturated 0.1 M KOH with a rotation rate of 1600 rpm. In KOD experiment, Ag/AgCl was used as reference electrode.

**Intrinsic current densities of various active molecules**

The values of intrinsic current densities of various active molecules containing pyridinic N species ($J_{\text{pyridinic N}}$) in ORR process were calculated by assuming that all pyridinic N groups are involved during ORR process.

$$J_{\text{pyridinic N}} = \Delta j / n$$

Where Δj is the difference values of model systems and OLC support at current densities at 0.2 $V_{RHE}$-1.0 $V_{RHE}$. The moles of the active molecules that are deposited onto the glass carbon disk are represented by n. Prior to the calculation of $J_{\text{pyridinic N}}$, the mass relevance fraction of pure active molecules is obtained from TG.

**Calculation of theoretical TOF and Faradaic efficiency**

The theoretical values of TOF at 1.6 $V_{RHE}$ were calculated by assuming that every nitrogen group is involved in the catalysis.

$$TOF = \frac{j \times S}{4 \times F \times n}$$

Where j is the measured current density at $\eta$ = 0.370 $V_{RHE}$ and S is the surface area of GCE (0.196 cm$^2$). F represents Faraday constant (96485.3 C mol$^{-1}$), and the number 4 originates from the four electron transfer. It means that 4 electrons/mol per produced $O_2$ are required. n is the moles of the pure active molecules that are deposited onto the glass carbon disk.

The Tafel slope was calculated according to the Tafel equation as follows:

$$\eta = b \cdot \log(\frac{j}{j_0})$$

where η denotes the overpotential, b denotes the Tafel slope, j denotes the current density, and $j_0$ denotes the exchange current density.

To investigate the reaction mechanism of OER, the RRDE voltammograms were conducted. A scan rate of 10 mV $s^{-1}$ and a rotation rate of 1600 rpm were applied for RRDE tests. Specifically, in order to determine the reaction pathway (electron transfer number) for OER by detecting the $HO_2^-$ formation, the ring potential was held constantly at 1.465 $V_{RHE}$ for oxidizing $HO_2^-$ intermediate in Ar-saturated 0.1 M KOH.

The Faradaic efficiency of the system was measured to ensure that the oxidation current derived from oxygen evolution rather than other side reactions. Here, the ring potential was held constantly at 0.465 $V_{RHE}$ to reduce the $O_2$ formed from the catalyst on the disk electrode in Ar-saturated 0.1 M KOH solution. During Faradaic efficiency measurements, the disc potential was held at 1.7 $V_{RHE}$ to allow the OER to proceed for 2 h. The Faradaic efficiency (FE) was determined as follows:

$$FE = \frac{I_r}{I_d N}$$

Where $I_r$ is the ring current density, $I_d$ is the current density of the disk electrode and N is the current collection efficiency (37%).

**Kinetic isotope effect (KIE) values**

KIE values are defined by the ratio of the isotopic rate constants ($k_0^H/k_0^D = k_H/k_D$). $k_H/k_D$ can be calculated by the exchange current densities on a catalyst in $H_2O$ ($j_0^H$) and $D_2O$ ($j_0^D$),[1] that is,

$$KIE = k_H/k_D = j_0^H C_0^D / j_0^D C_0^H$$

where $C_0$ is the $O_2$ concentration in $H_2O$ or $D_2O$. The value of $C_0^D/C_0^H$ is defined to be 1.101 at 298 K. Exchange current densities ($j_0$) can be calculated by

$$j = j_0 Exp(n\alpha F\eta / RT)$$

where n, α, F, R, and T denote the number of electrons involved in the electrode reaction number, anodic charge transfer coefficient, Faraday constant, gas constant (8.314 J. $K^{-1}.mol^{-1}$), and reaction temperature (298K), respectively. The α can be calculated from the Tafel slope b (V $dec^{-1}$), that is

$$b = 2.303RT / n\alpha F$$

And thus, $KIE = k_H/k_D = 1.101 \times \frac{j_H}{j_D} \times \frac{Exp(n\alpha_D F\eta / RT)}{Exp(n\alpha_H F\eta / RT)}$

Here, Tafel slopes of DBAD+OLC for ORR process in KOH are 0.110 V $dec^{-1}$ and 0.056 V $dec^{-1}$ at 0.6 $V_{RHE}$ and 0.7 $V_{RHE}$ (**Figure S28**), respectively. Tafel slopes of DBAD+OLC in KOD are 0.113 V $dec^{-1}$ and 0.058 V $dec^{-1}$ at 0.6 $V_{RHE}$ and 0.7 $V_{RHE}$, respectively. And thus KIE values of DBAD+OLC are calculated to be 1.45~1.61 at 0.6 $V_{RHE}$~0.7 $V_{RHE}$. In the case of DBAD+OLC for OER process in KOH, its Tafel slope is 0.076 V $dec^{-1}$. Tafel slope of DBAD+OLC in KOD is 0.086 V $dec^{-1}$. And thus KIE values of DBAD+OLC are calculated to be 1.51~1.68 at 1.567 $V_{RHE}$~1.60 $V_{RHE}$.

[1] Sakaushi, K.; Eckardt, M.; Lyalin, A.; Taketsugu, T.; Behm, R. J.; Uosaki, K. Microscopic electrode processes in the four-electron oxygen reduction on highly active carbon-based electrocatalysts. *ACS Catal.* **2018**, *8*, 8162-8176.

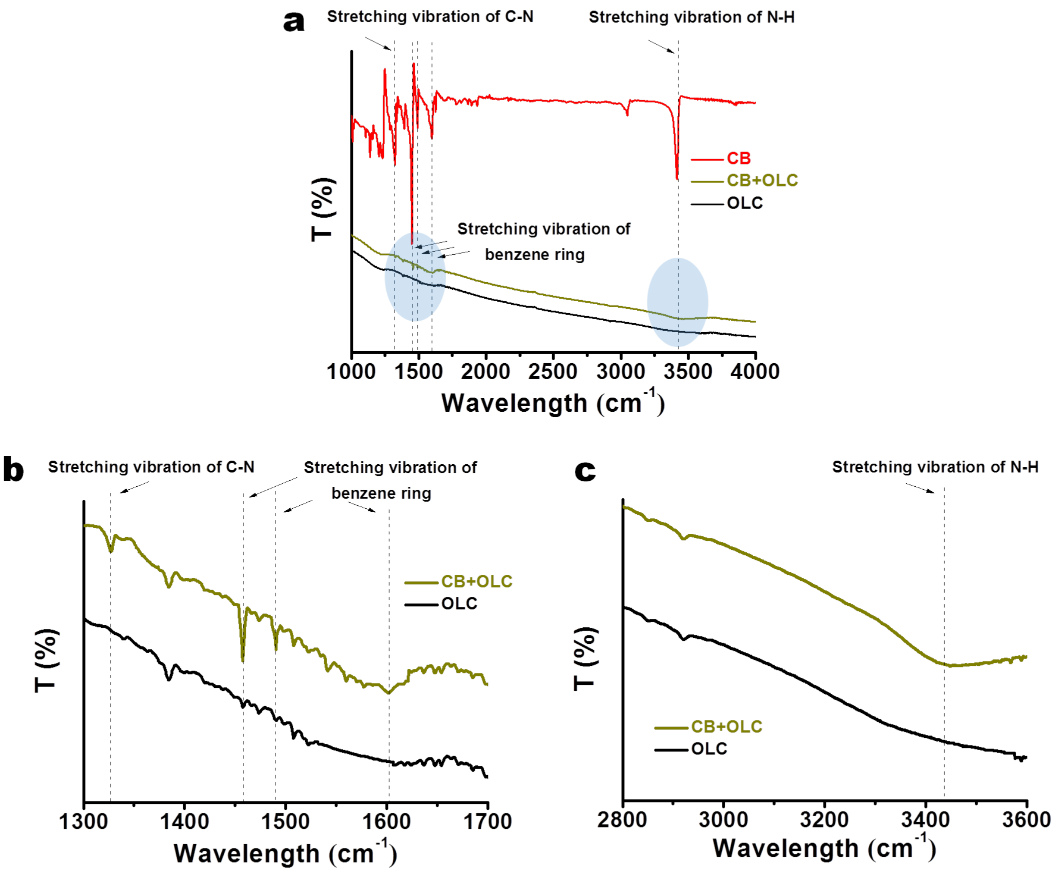


**Figure S1. Attenuated total reflectance infrared (ATR-IR) spectra of pure CB, OLC and CB+OLC samples. (a)** Full views of ATR-IR spectra. **(b)-(c)** Enlarged views of ATR-IR spectra at 1300-1700 $cm^{-1}$ and 2800-3600 $cm^{-1}$ regions, respectively.

As shown in Figure S1, the main characteristic peaks of pure CB powder located at 3414$cm^{-1}$ and 1322 $cm^{-1}$ belong to stretching vibration of the N-H and the C-N groups, respectively. The peaks located at 1600 $cm^{-1}$, 1488 $cm^{-1}$ and 1450 $cm^{-1}$ are designed as vibrations of aromatic rings. It is clearly suggested that the CB molecules from the obtained CB+OLC sample also show the main characteristic peaks of N-H, C-N groups and aromatic rings. The slight shift of peaks can be attributed to the influence of π-π interaction between CB molecules and OLC support. The measurements suggest that the molecular structure of CB is not destroyed during the preparation process.

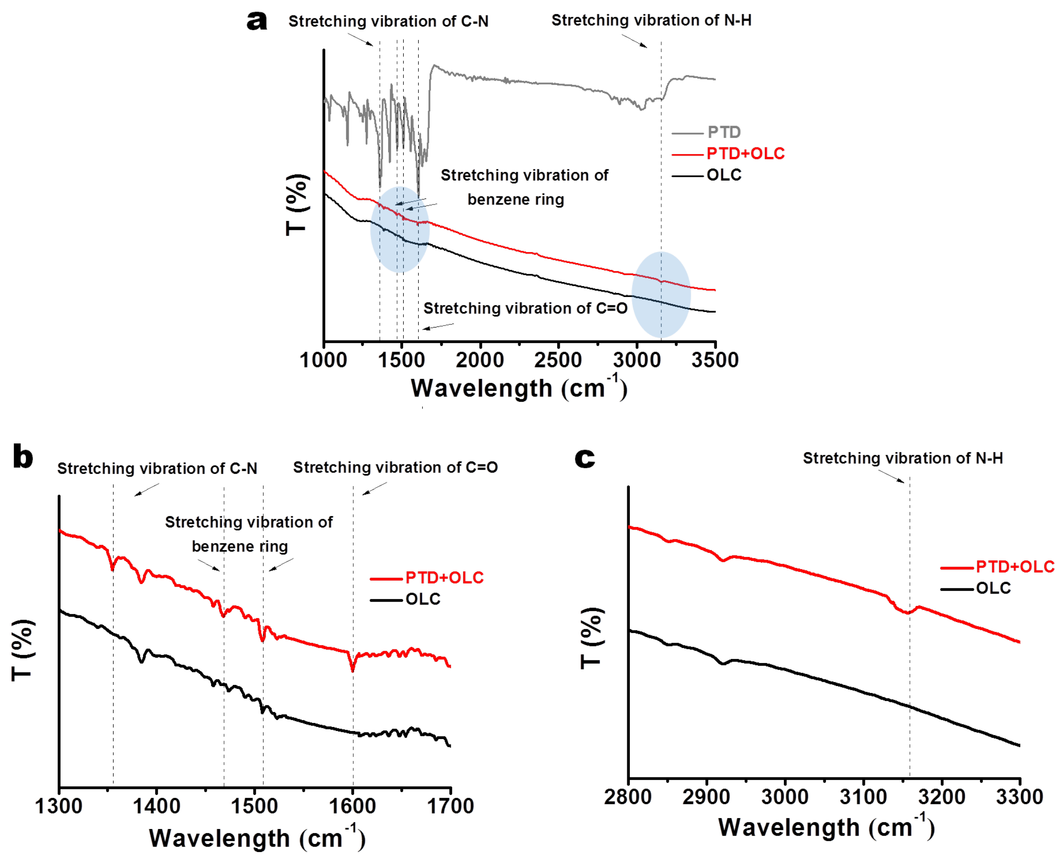


**Figure S2. ATR-IR spectra of pure PTD, OLC and PTD+OLC samples. (a)** Full views of ATR-IR spectra. **(b)-(c)** Enlarged views of ATR-IR spectra at 1300-1700 $cm^{-1}$ and 2800-3300 $cm^{-1}$ regions, respectively.

As shown in Figure S2, the main characteristic peaks of pure PTD powder located at 3159 $cm^{-1}$, 1603 $cm^{-1}$ and 1353 $cm^{-1}$ are ascribed as stretching vibrations of the N-H, the C=O and the C-N groups, respectively. The peaks located at 1509 $cm^{-1}$ and 1474 $cm^{-1}$ are assigned as vibrations of aromatic rings. It can be seen that the PTD molecules also show the main characteristic peaks of N-H, C=O, C-N groups and aromatic rings over PTD+OLC sample. The slight shift of peaks can be attributed to the influence of π-π interaction between PTD molecules and OLC support. The measurements indicate that the molecular structure of PTD is not destroyed during the preparation process.

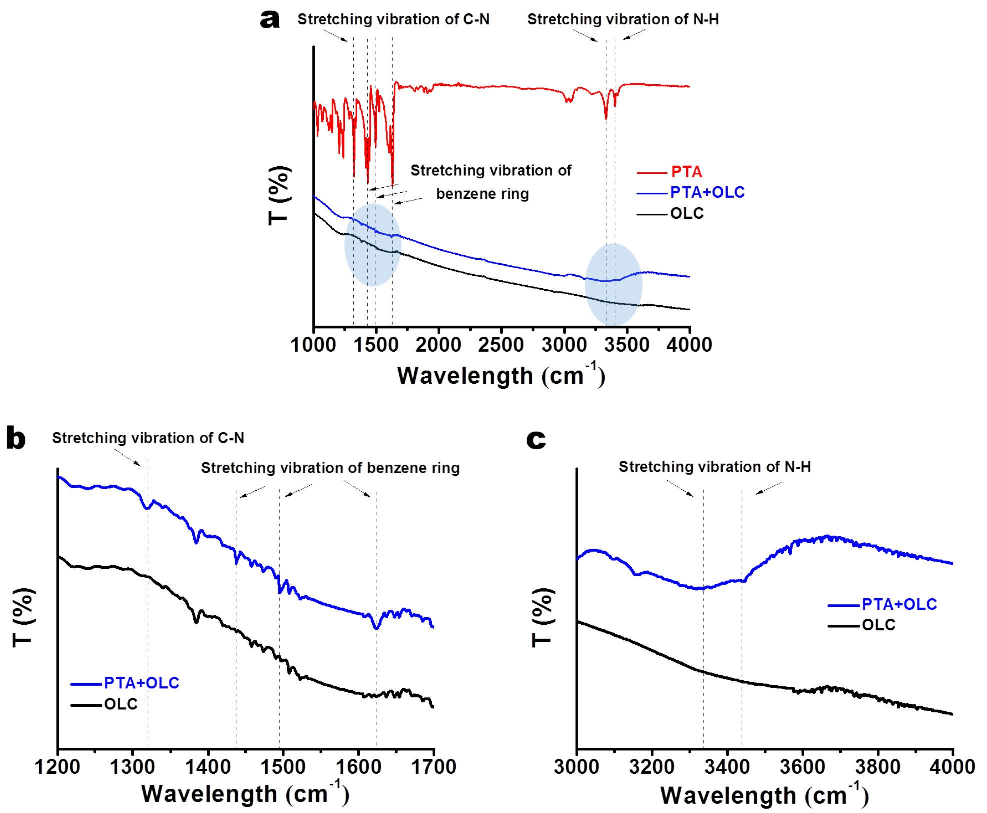


**Figure S3. ATR-IR spectra of pure PTA, OLC and PTA+OLC samples. (a)** Full views of ATR-IR spectra. **(b)-(c)** Enlarged views of ATR-IR spectra at 1200-1700 $cm^{-1}$ and 3000-4000 $cm^{-1}$ regions, respectively.

As shown in Figure S3, the main characteristic peak of pure PTA powder located at 1321 $cm^{-1}$ is designated as stretching vibration of the C-N group. The peaks located at 3407 $cm^{-1}$ and 3336 $cm^{-1}$ belong to stretching vibration of the N-H group. The peaks located at 1631 $cm^{-1}$, 1497 $cm^{-1}$ and 1434 $cm^{-1}$ are ascribed as vibrations of aromatic rings. It is clearly shown that the PTA molecules over PTA+OLC sample possess the main characteristic peaks of aromatic rings, N-H and C-N groups. The slight shift of peaks can be attributed to the influence of $\pi$-$\pi$ interaction between PTA molecules and OLC support. The measurements confirm that the molecular structure of PTA is not destroyed during the preparation process.

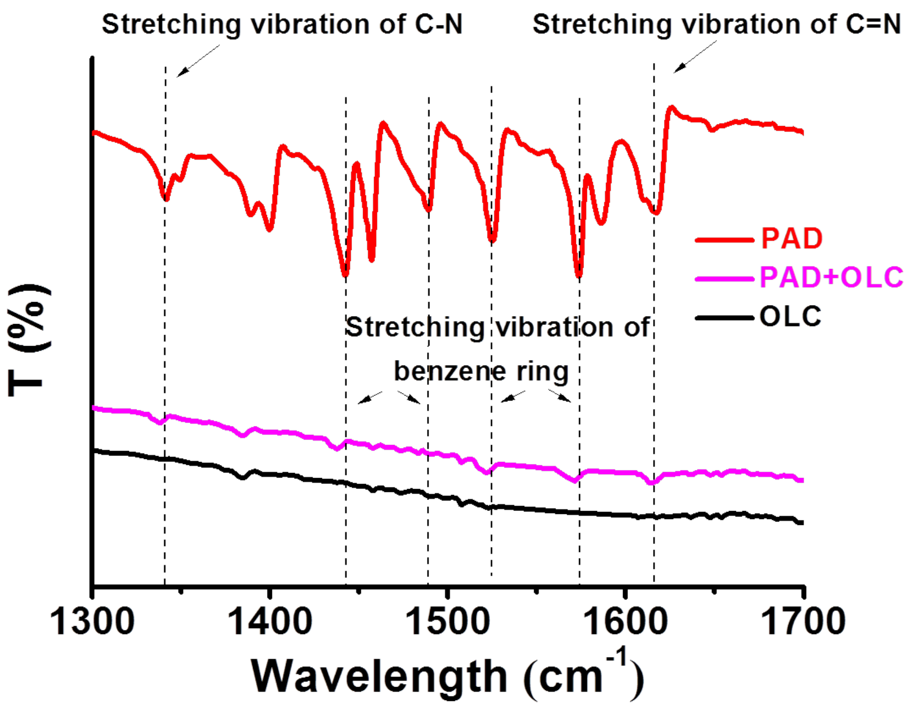


**Figure S4.** ATR-IR spectra of pure PAD, OLC and PAD+OLC samples.

As shown in Figure S4, the main characteristic peaks of pure PAD powder located at 1617 $cm^{-1}$ and 1341 $cm^{-1}$ are designated as stretching vibrations of the C=N and the C-N groups, respectively. The peaks located at 1574 $cm^{-1}$, 1529 $cm^{-1}$, 1489 $cm^{-1}$and 1442 $cm^{-1}$ are ascribed as vibrations of the aromatic rings. It is clearly seen that the PAD molecules on PAD+OLC sample display the main characteristic peaks of C=N, C-N groups and aromatic rings. The slight shift of peaks can be attributed to the influence of π-π interaction between PAD molecules and OLC support. The measurements confirm that the molecular structure of PAD is not destroyed during the preparation process.

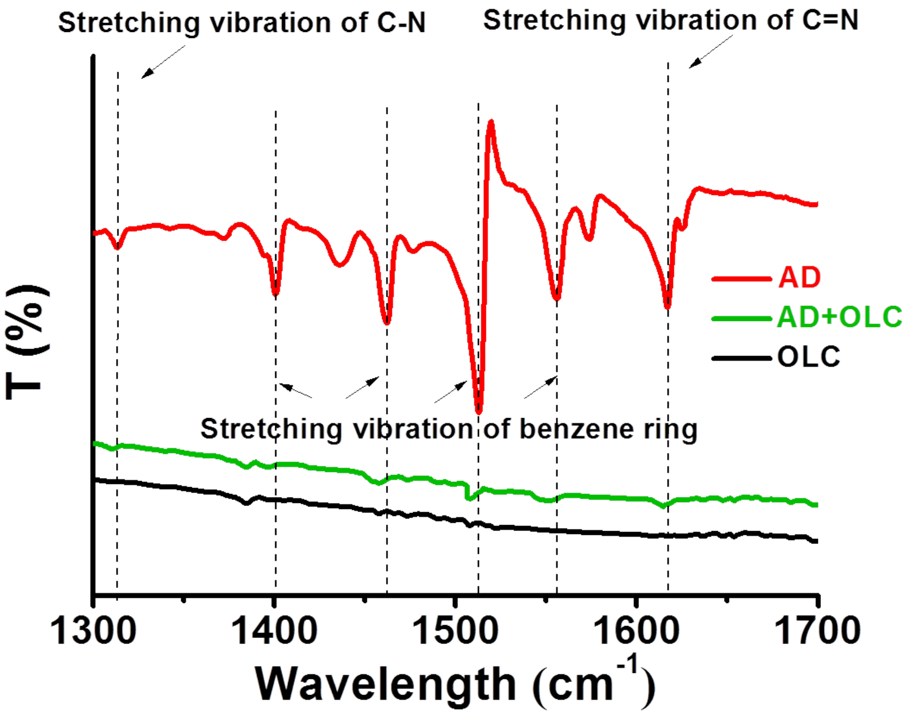


**Figure S5.** ATR-IR spectra of pure AD, OLC and AD+OLC samples.

As shown in Figure S5, the main characteristic peaks of pure AD powder located at 1617 $cm^{-1}$ and 1371 $cm^{-1}$ belong to stretching vibrations of the C=N and the C-N groups, respectively. The peaks located at 1556 $cm^{-1}$, 1512 $cm^{-1}$, 1462 $cm^{-1}$ and 1401 $cm^{-1}$ are ascribed as vibrations of the aromatic rings. It is clearly observed that the AD molecules over AD+OLC sample have the main characteristic peaks of C=N, C-N groups and aromatic rings. The slight shift of peaks can be attributed to the influence of π-π interaction between AD molecules and OLC support. The measurements indicate that the molecular structure of AD is not destroyed during the preparation process.

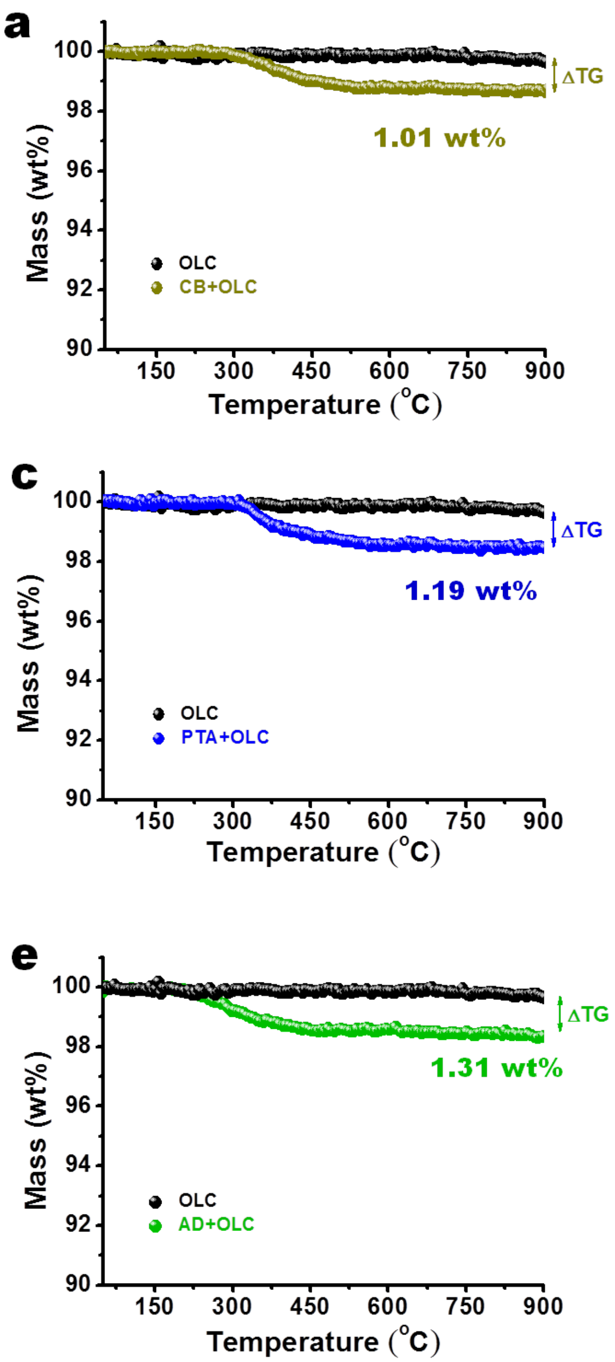


**Figure S6.** TG analysis of pure OLC, CB+OLC **(a)**, PTD+OLC **(b)**, PTA+OLC **(c)**, PAD+OLC **(d)**, AD+OLC **(e)** and AD+OLC-1, AD+OLC-2 **(f)** model catalytic system. The active molecules can be quantified by the difference between remaining mass of pure OLC and remaining mass of supported model catalysts.

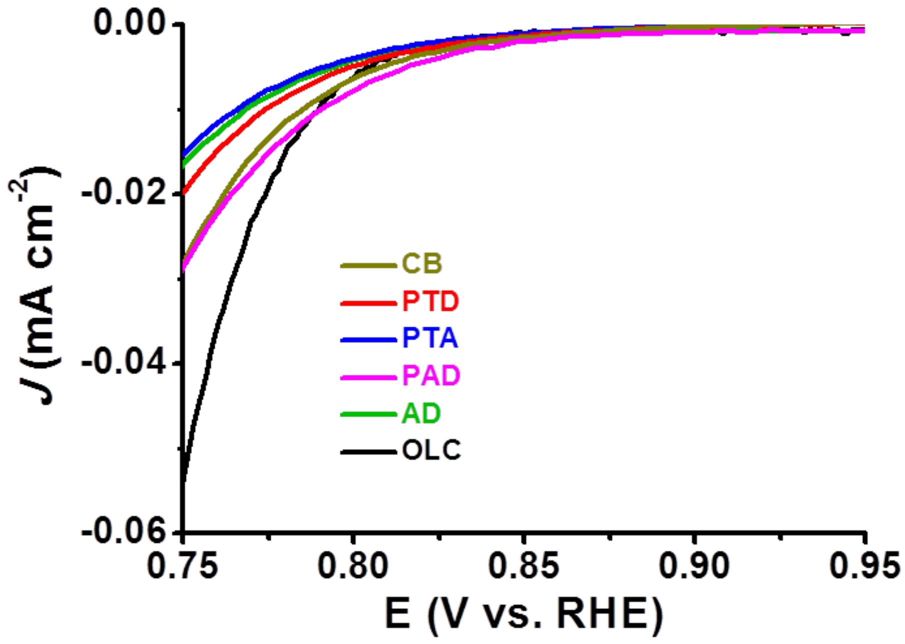


**Figure S7**. **Electrochemical measurements of various pure N-containing organic molecules and pristine OLC catalysts for the ORR in 0.1 M KOH.** The loading of each sample on the glassy carbon electrode is 0.051 mg/$cm^2$.

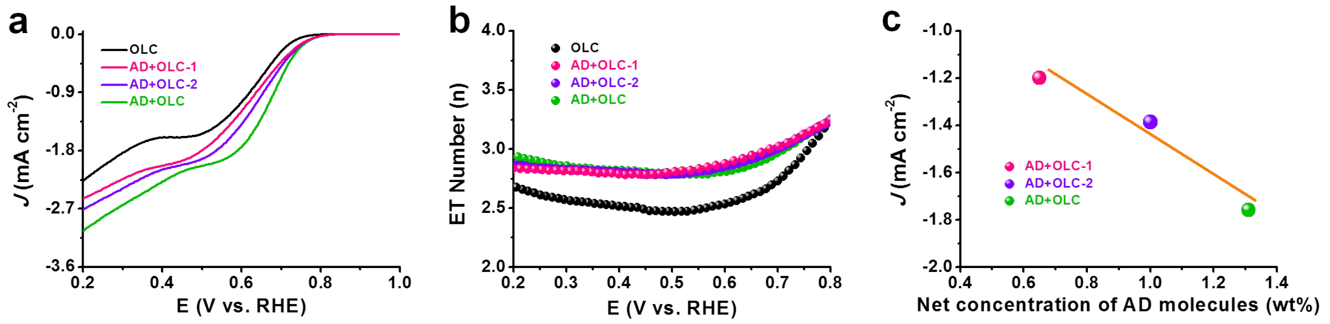


**Figure S8**. **Electrochemical measurements of AD+OLC with different concentrations of AD molecules for the ORR in 0.1 M KOH.** (a) LSV curves. (b) Electron transfer (ET) numbers based on RRDE measurements. (c) Relationship between the net concentration of AD molecules and current density at 0.6 $V_{RHE}$. The net concentration of AD molecules in AD+OLC catalyst is obtained with TG.

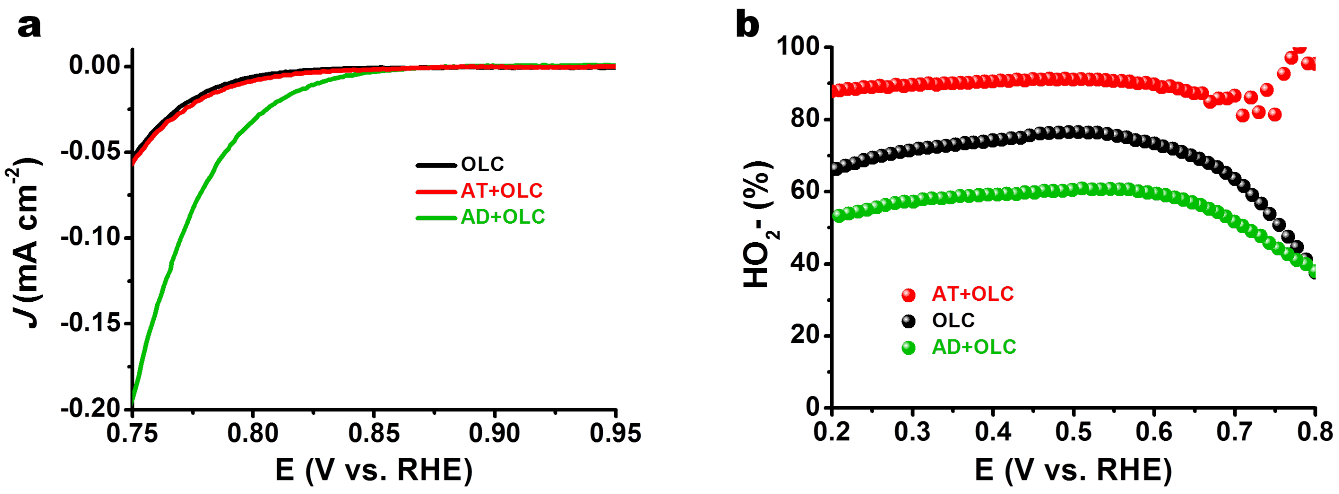


**Figure S9**. **Electrochemical measurements of OLC, supported anthracene (AT+OLC), supported AD (AD+OLC) model catalysts for the ORR in 0.1 M KOH.** The loading of each samples on the glassy carbon electrode is 0.051 mg/cm$^2$. Here, AT was used as a model molecule to mimic zigzag configuration of carbon materials.

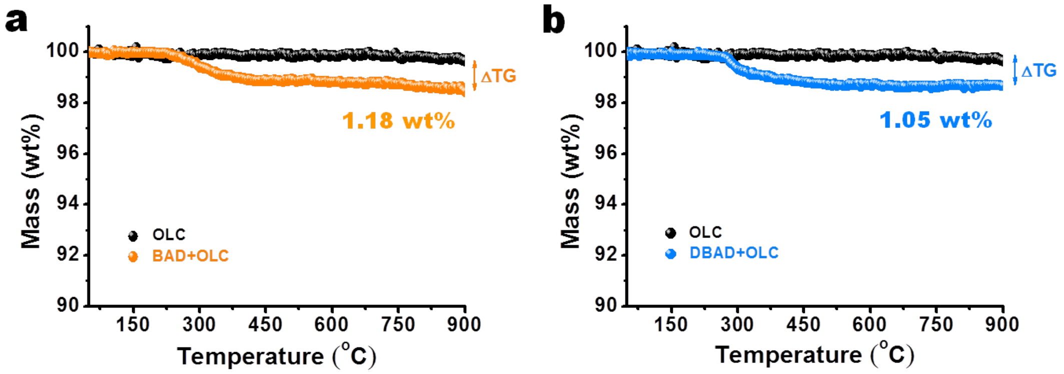


**Figure S10.** TG analysis of pristine OLC, BAD + OLC **(a)** and DBAD + OLC **(b)** catalysts**.** The active molecules can be quantified by using remaining mass of pure OLC minus remaining mass of supported model catalysts.

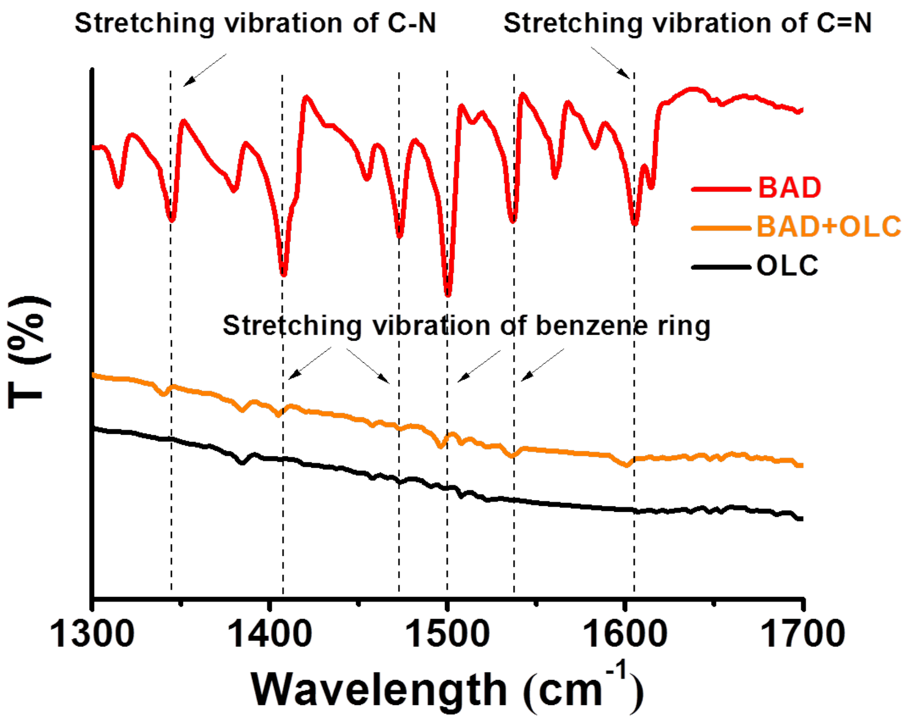


**Figure S11.** ATR-IR spectra of pure BAD, OLC and BAD+OLC samples.

As shown in Figure S11, the main characteristic peaks of pure PAD powder located at 1604 $cm^{-1}$ and 1347 $cm^{-1}$ are attributed to stretching vibrations of the C=N and the C-N groups, respectively. The peaks located at 1537 $cm^{-1}$, 1500 $cm^{-1}$, 1472 $cm^{-1}$ and 1407 $cm^{-1}$ are ascribed as vibrations of the aromatic rings. It is clearly displayed that the BAD molecules over BAD+OLC sample have the main characteristic peaks of C=N, C-N groups and aromatic rings. The slight shift of peaks can be attributed to the influence of $\pi$-$\pi$ interaction between BAD molecules and OLC support. The measurements confirm that the molecular structure of BAD is not destroyed.

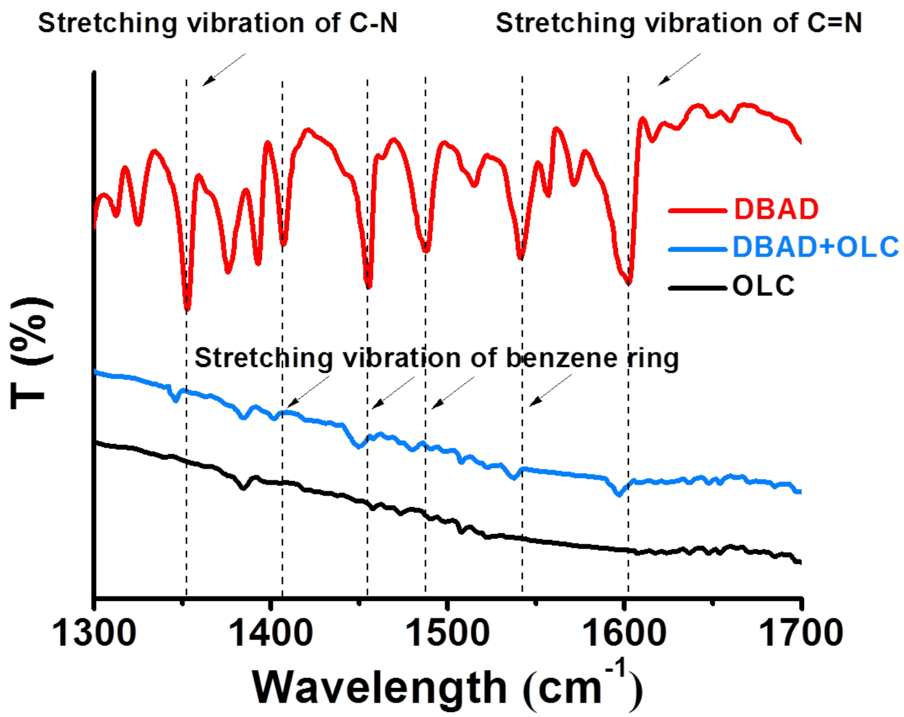


**Figure S12.** ATR-IR spectra of pure DBAD, OLC and DBAD+OLC samples.

As shown in Figure S12, the main characteristic peaks of pure DBAD powder located at 1601 $cm^{-1}$ and 1357 $cm^{-1}$ are assigned to stretching vibrations of the C=N and the C-N groups, respectively. The peaks located at 1543 $cm^{-1}$, 1487 $cm^{-1}$, 1454 $cm^{-1}$ and 1406 $cm^{-1}$ belong to vibrations of the aromatic rings. It is clearly seen that the DBAD molecules over DBAD+OLC sample possess the main characteristic peaks of C=N, C-N groups and aromatic rings. The slight shift of peaks can be attributed to the influence of $\pi$-$\pi$ interaction between DBAD molecules and OLC support. The measurements imply that the molecular structure of DBAD is not destroyed during the preparation process.

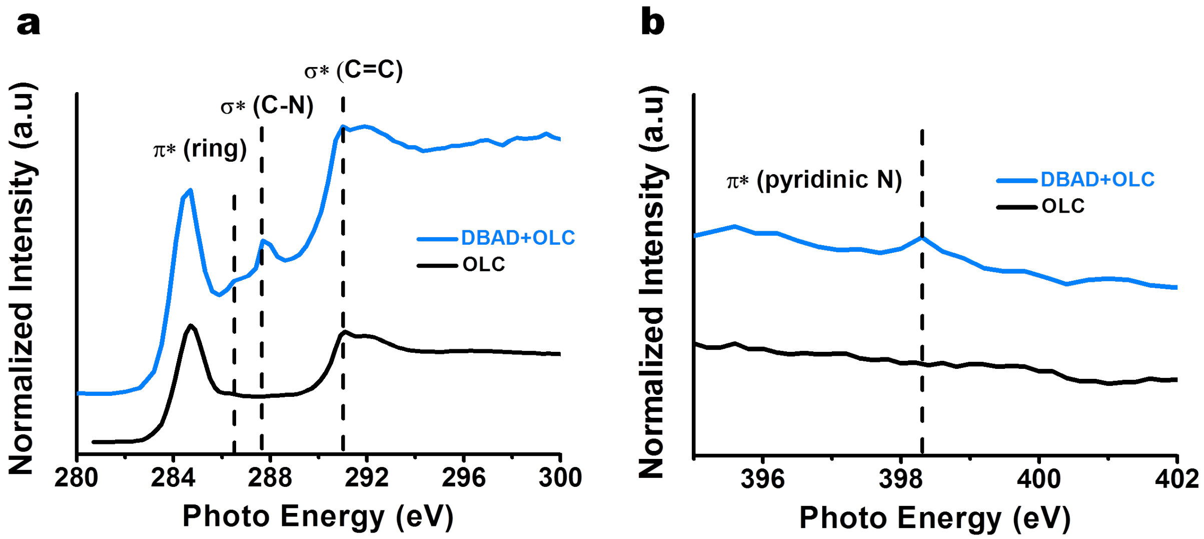


**Figure S13. X-ray absorption near-edge structure (XANES) spectra (incident angle θ=45º) of representative pristine OLC, DBAD+OLC catalysts.** (**a**) C K-edge and (**b**) N K-edge.

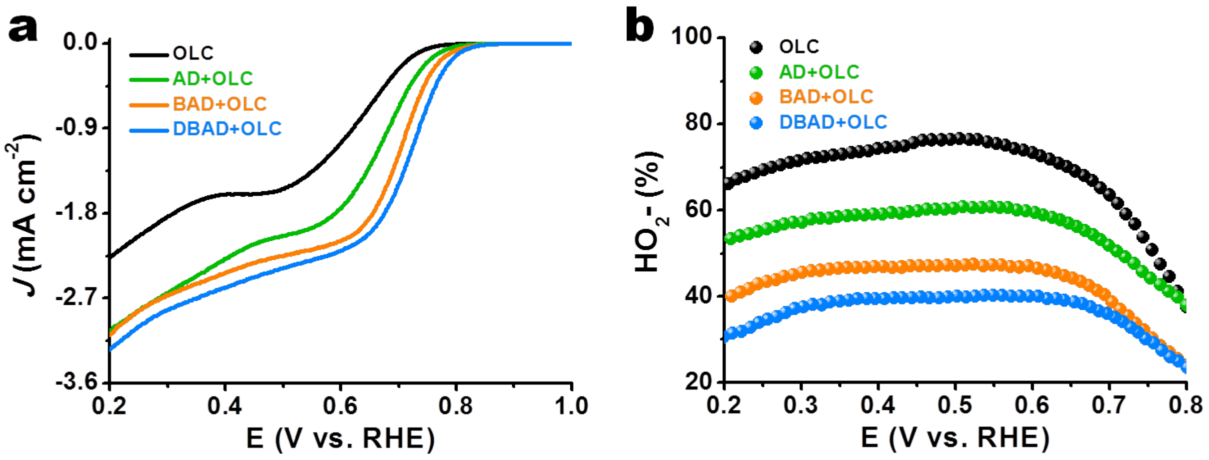


**Figure S14**. **Electrochemical measurements of model catalytic systems consisting of pyridinic N-containing organic molecules and OLC support for the ORR in 0.1 M KOH.** The loading of each samples on the glassy carbon electrode is 0.051 mg/cm$^2$. (**a**) LSV curves. (**b**) $HO_2$- selectivities based on RRDE measurements.

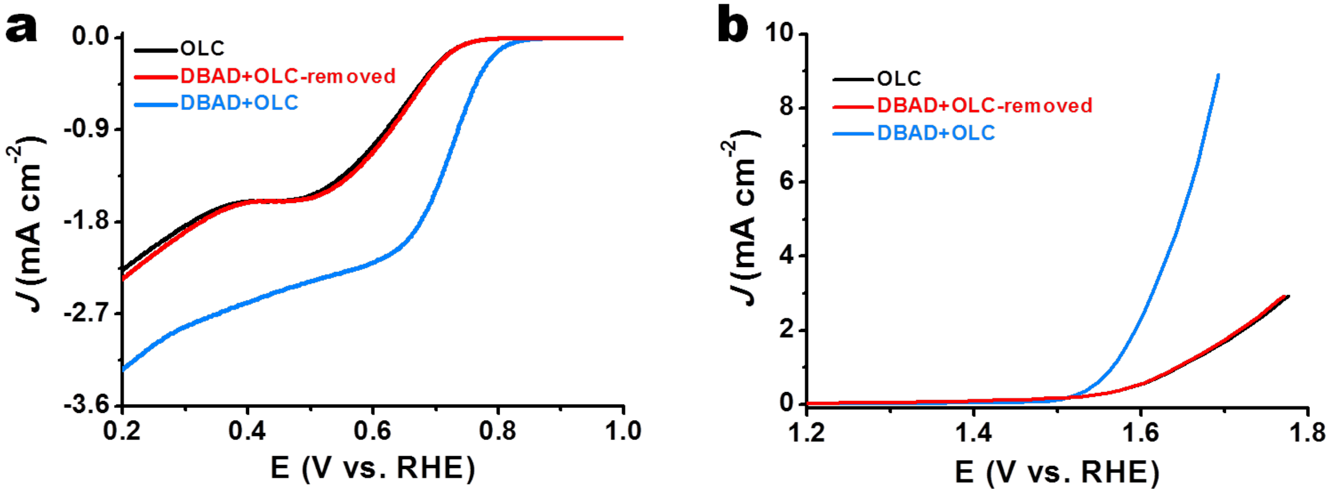


**Figure S15**. **Catalytic activity of pure OLC, DBAD+OLC and removed DBAD+OLC catalysts.** Here, the removal of DBAD molecules was achieved by sonicating DBAD+OLC for 12 h in cyclohexane.

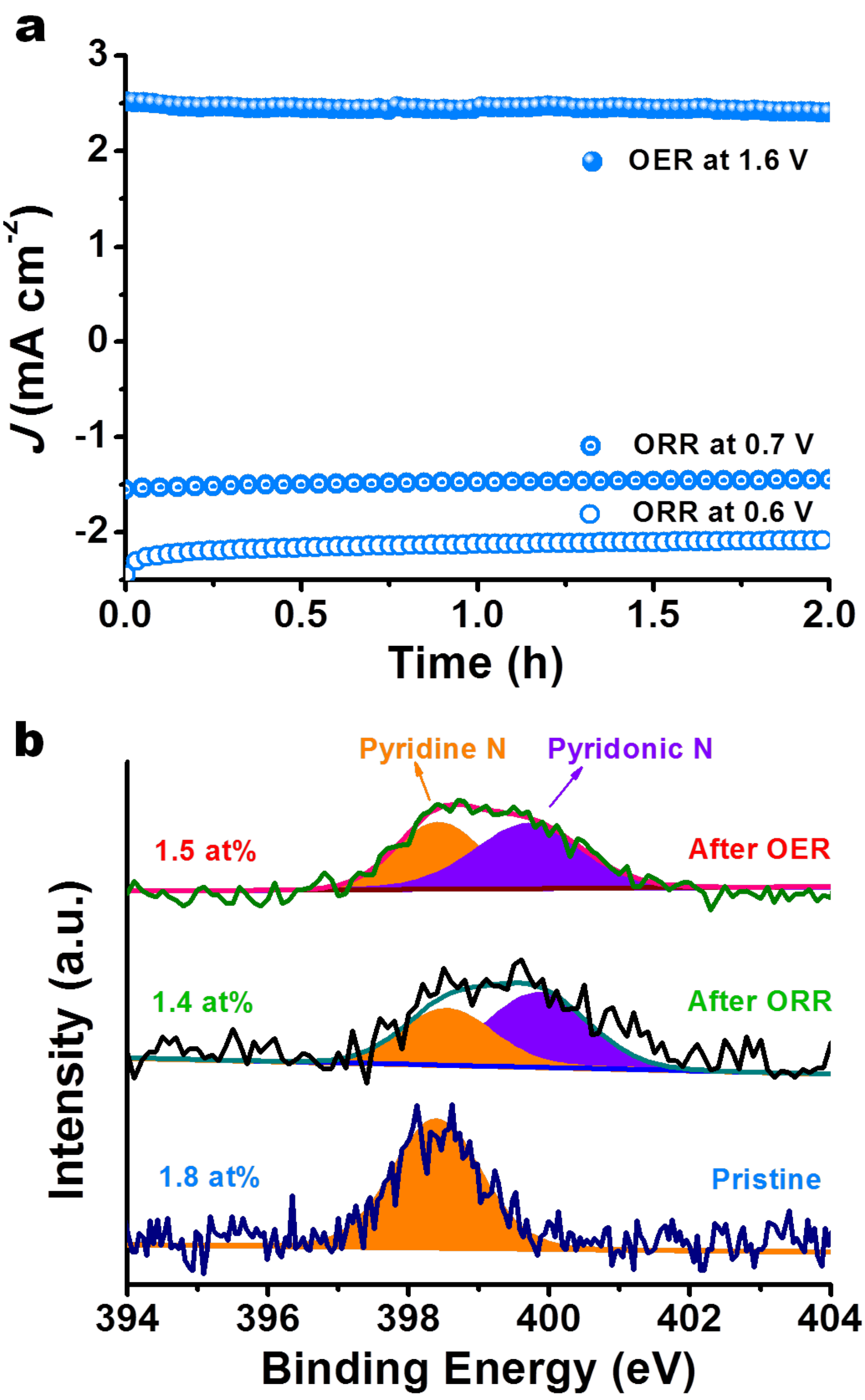


**Figure S16. Stability tests and XPS spectra of DBAD+OLC catalyst.** (**a**) At constant potentials for ORR and OER processes. (**b**) Measured before and after different reaction processes.

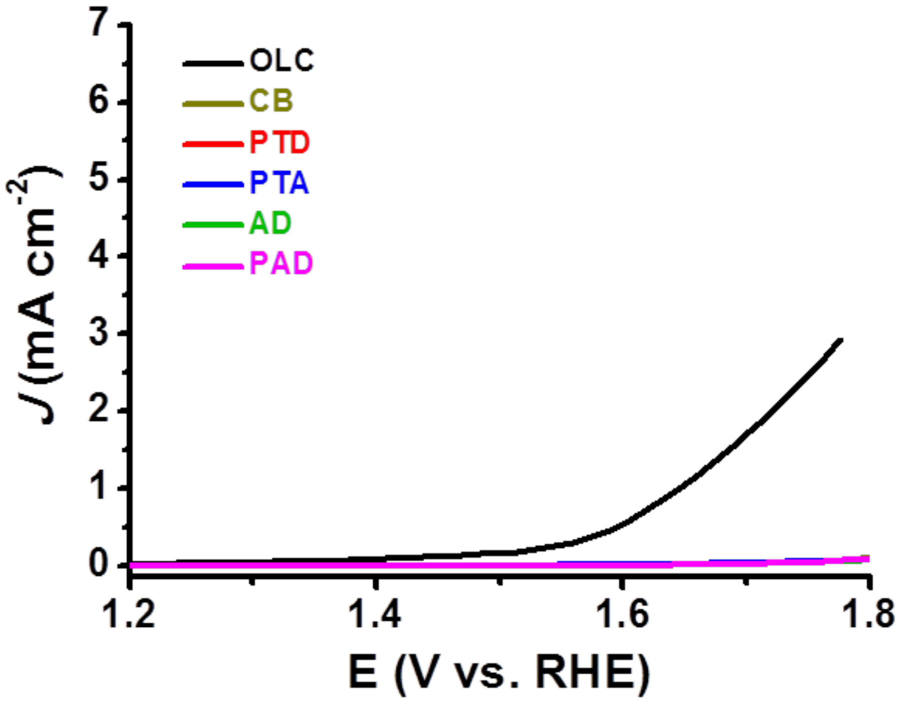


**Figure S17**. LSV curves of pure N-containing organic molecules and pure OLC for the OER in 0.1 M KOH.

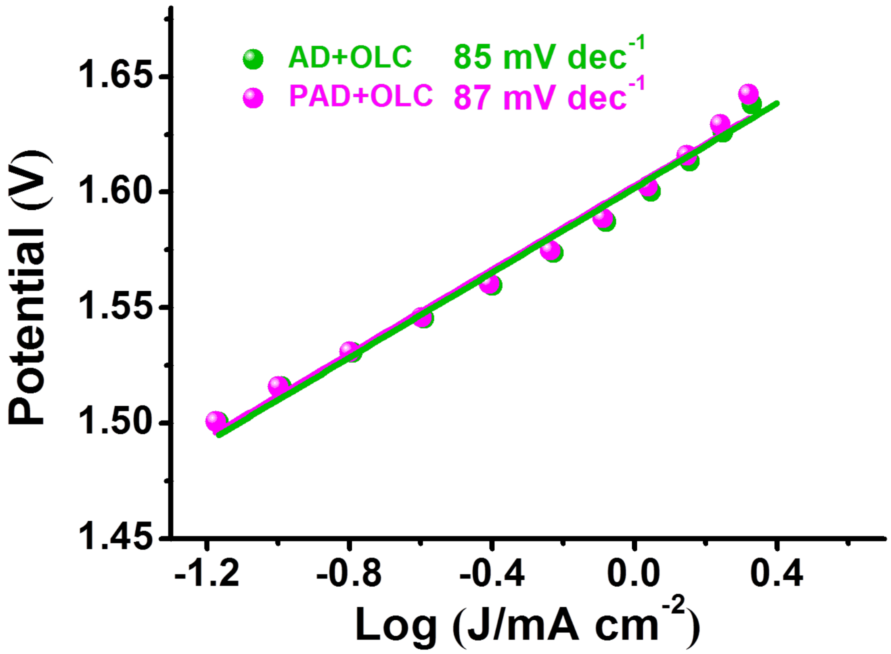


**Figure S18. Tafel slopes of AD+OLC and PAD+OLC catalysts at a low working potential region.** Here, pyridinic N species of AD molecule locates at zigzag edge position, while pyridinic N species of PAD molecule locates at armchair edge position.

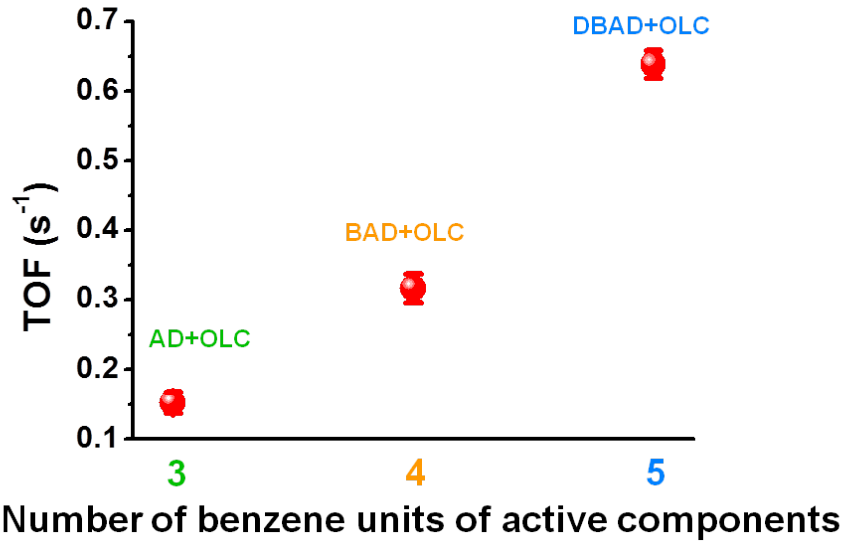


**Figure S19**. Number of benzene units of active components with single pyridinic N species as a function of theoretical TOF at 1.6 $V_{RHE}$.

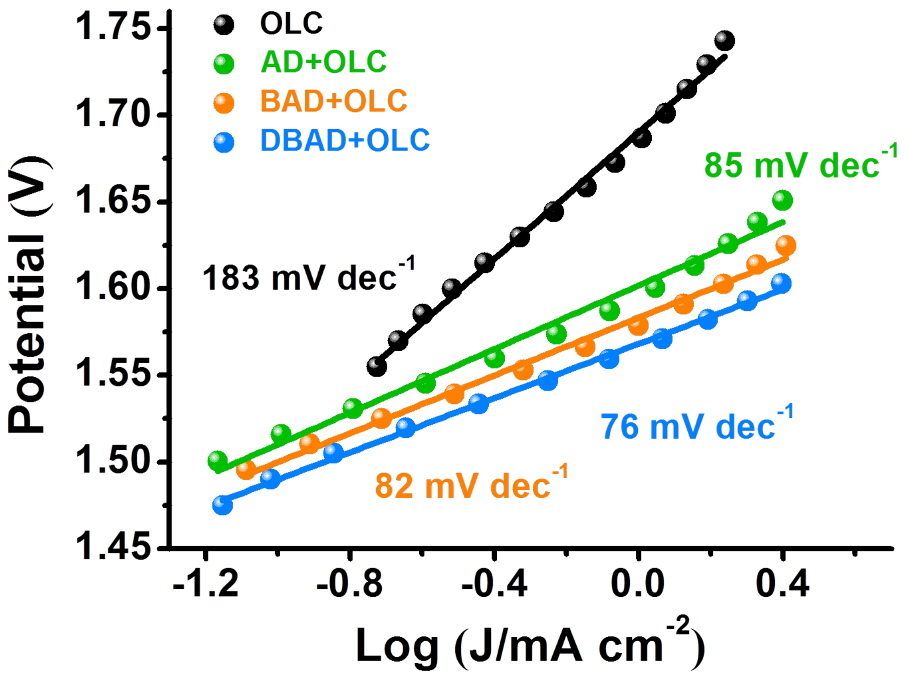


**Figure S20**. Tafel slopes of model catalytic systems consisting of pyridinic N-containing organic molecules and OLC support at a low working potential region.

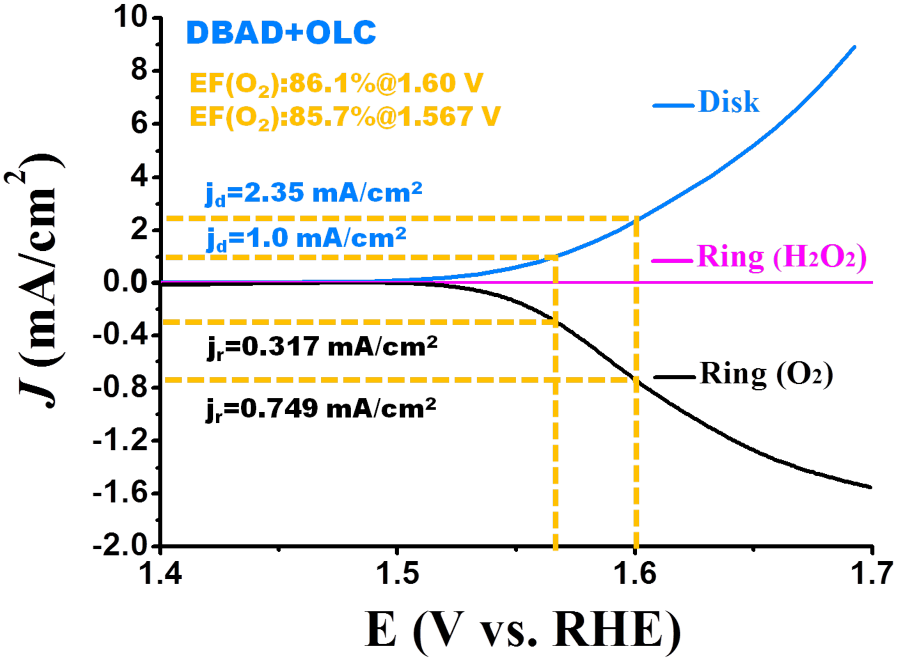


**Figure S21**. **Detection of $H_2O_2$ and $O_2$ evolution from DBAD+OLC catalyst applying RRDE measurements.** The Pt ring was biased at 1.465 V versus RHE to collect $H_2O_2$ and fixed at 0.465 V versus RHE to collect $O_2$ in Ar-saturated 0.1 M KOH. The current collection efficiency is 37%.

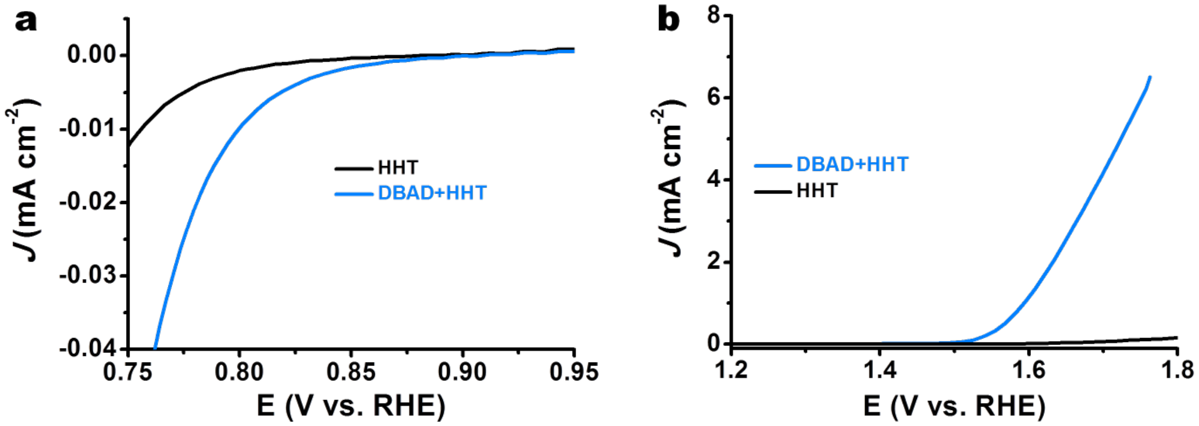


**Figure S22**. **Electrochemical activities of DBAD+HHT and pure HHT catalysts.** (**a**) ORR and (**b**) OER in 0.1 M KOH medium.

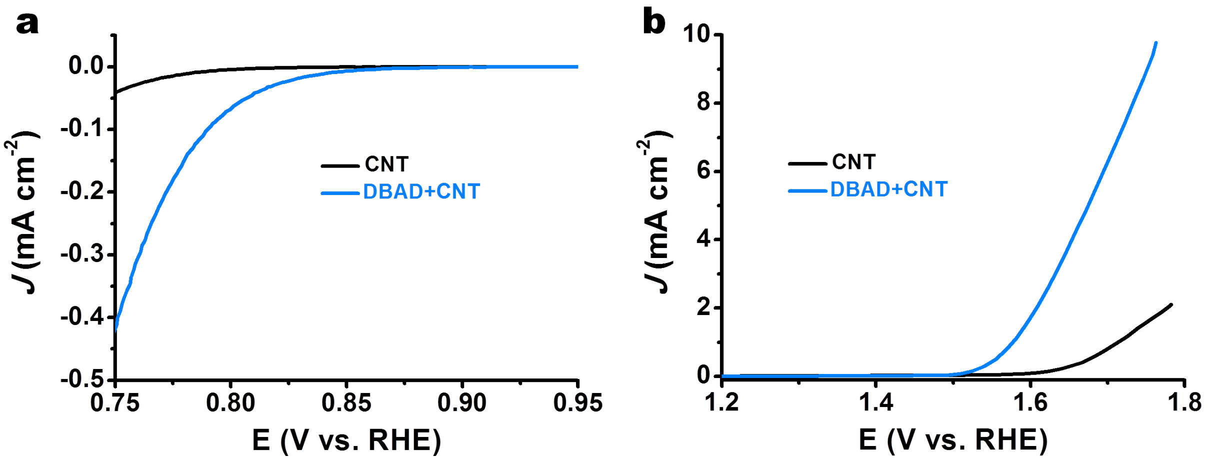


**Figure S23**. **Electrochemical activities of DBAD+CNT and pure CNT catalysts.** (**a**) ORR and (**b**) OER in 0.1 M KOH medium.

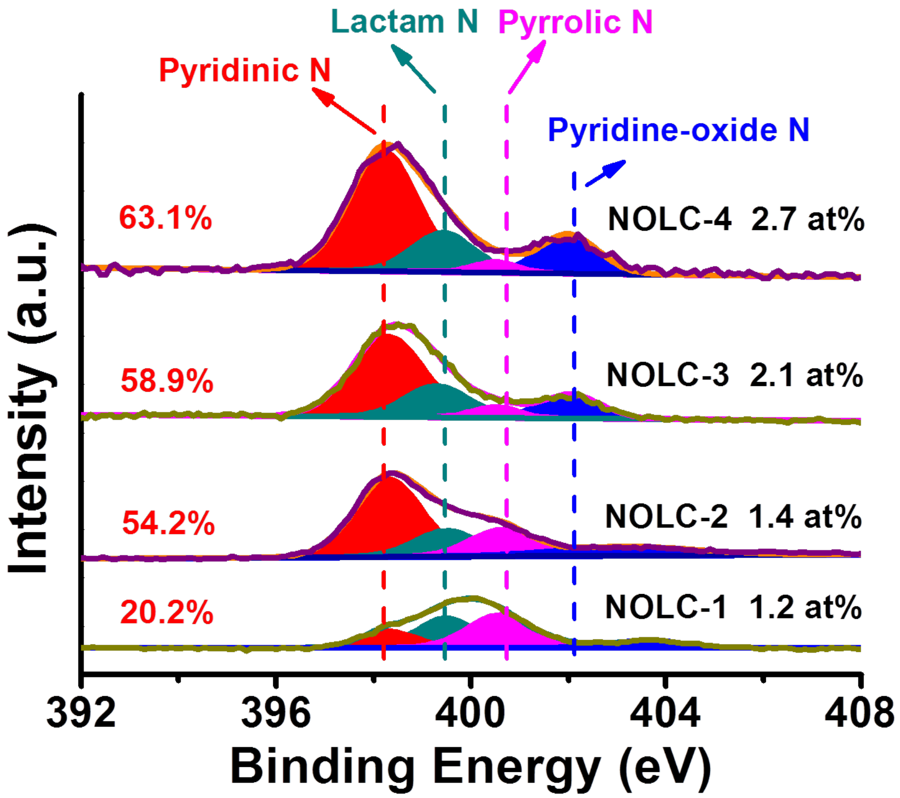


**Figure S24**. XPS spectra of various N-doped OLC (NOLC) catalysts without graphitic N (GN) species.

As shown in Figure S24, the N 1s spectra can be deconvoluted into the following four peaks: N1 (398.3 eV), N2 (399.3 eV), N3 (400.5 eV) and N4 (402~405 eV), which are assigned to pyridinic N, lactam or amine N (-O=C-NH or $-NH_2$), pyrrolic N and pyridine N-oxide, respectively. Here, no graphitic N (GN, ~401.3 eV) species can be observed in four N-doped OLC (NOLC). Among all nitrogen species, pyridinic N species exist in a high proportion with 20.2%~63.1%. The total contents of nitrogen species range from 1.2 at% to 2.7 at%.

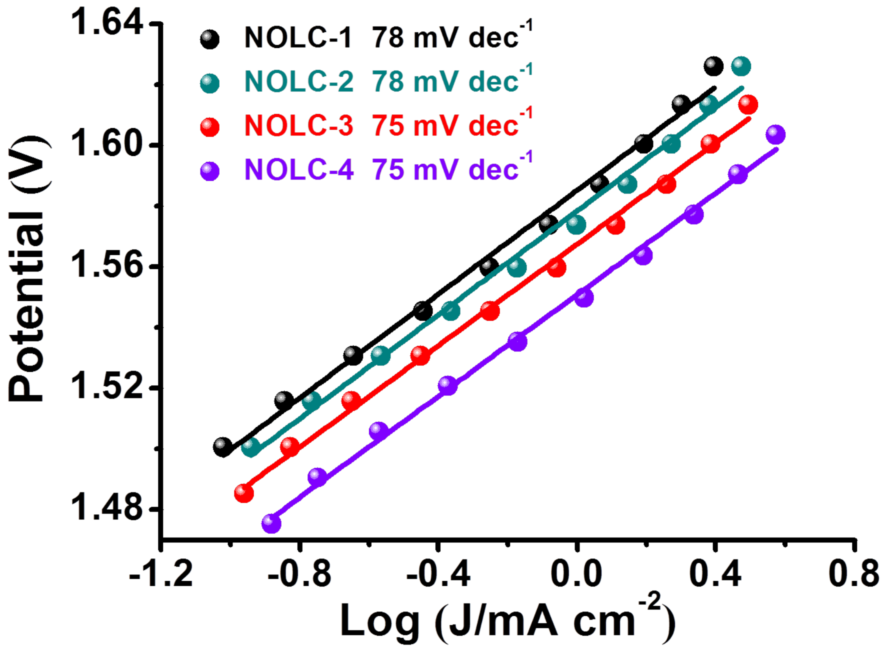


**Figure S25**. Tafel slopes of various NOLC catalysts at a low working potential region.

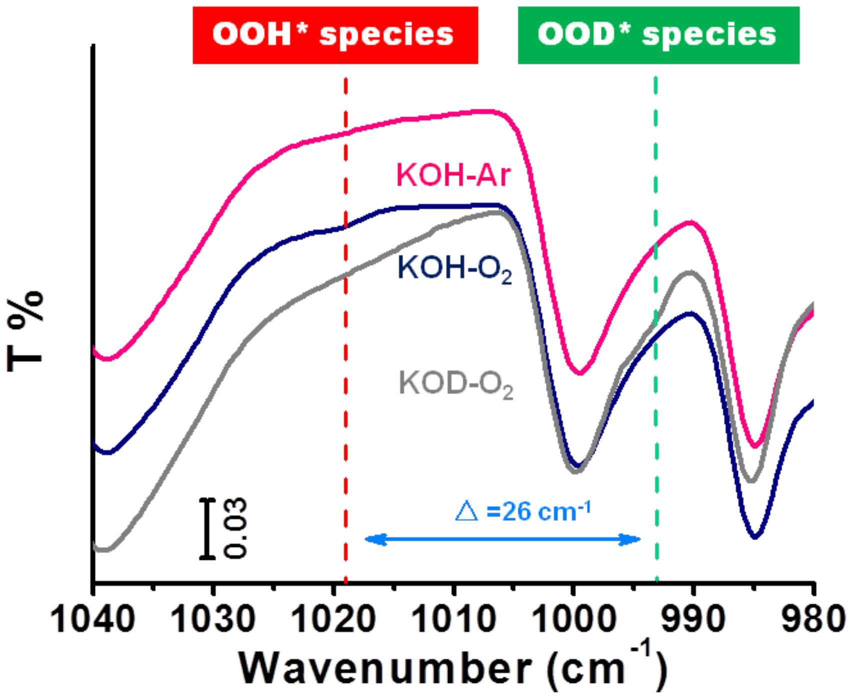


**Figure S26**. *In situ* ATR-IR spectra monitoring for OOH* and OOD* species at a specific range (980 $cm^{-1}$-1040 $cm^{-1}$) during the ORR process.

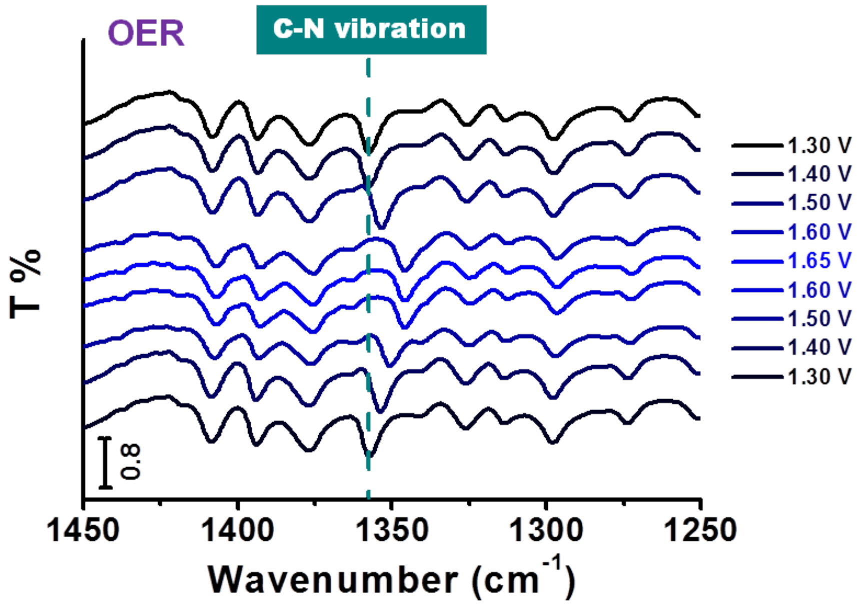


**Figure S27**. *In situ* ATR-IR spectra monitoring for C-N species of DBAD active component during the OER process.

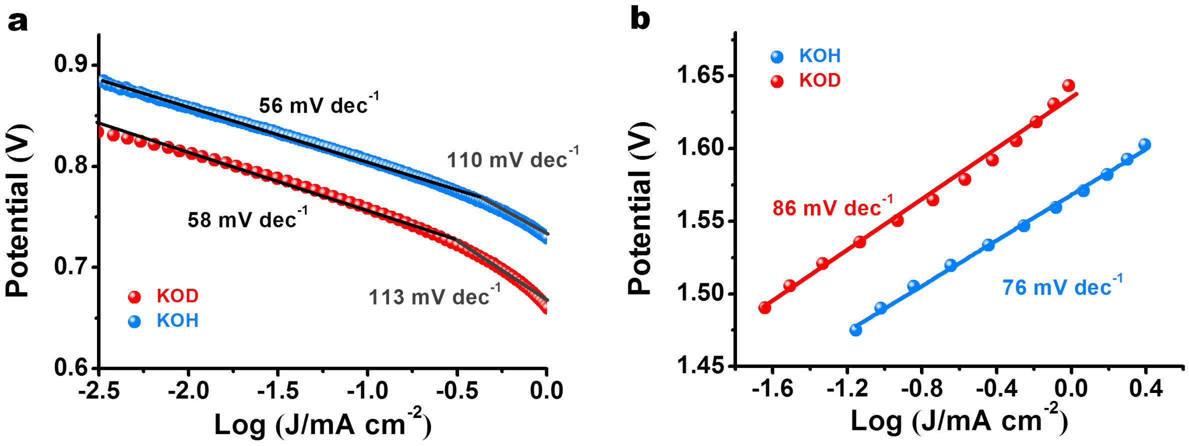


**Figure S28**. **Tafel slopes of DBAD+OLC catalyst.** (**a**) ORR and (**b**) OER processes at a low working potential region, respectively.

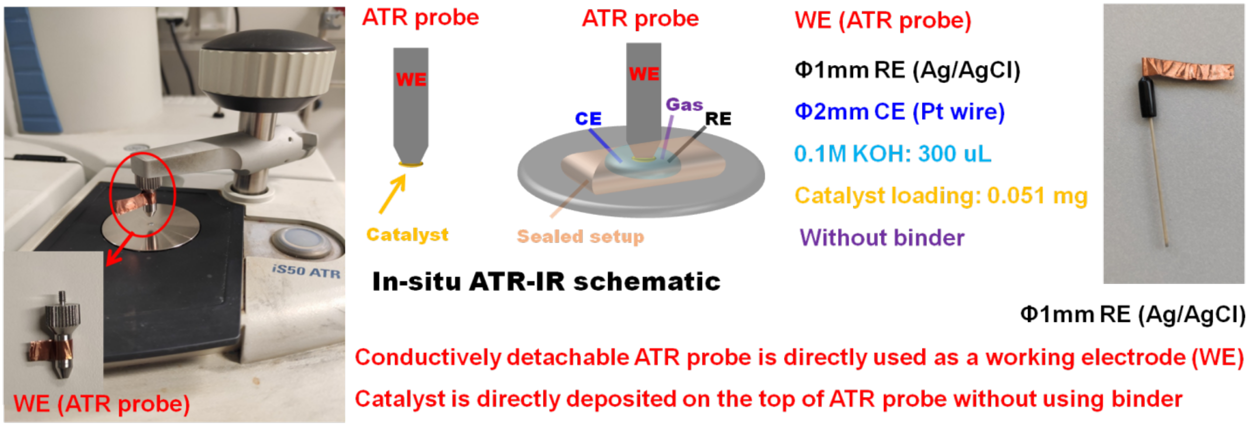


**Figure S29**. The in-situ ATR-IR schematic.
